# Spartalizumab in combination with platinum-doublet chemotherapy with or without canakinumab in patients with PD-L1-unselected, metastatic NSCLC

**DOI:** 10.1186/s12885-024-12841-2

**Published:** 2024-10-24

**Authors:** Armando Santoro, Garrido Pilar, Daniel S.W. Tan, Jon Zugazagoitia, Frances A. Shepherd, Alessandra Bearz, Fabrice Barlesi, Tae Min Kim, Tobias R. Overbeck, Enriqueta Felip, Can Cai, Simantini Eddy, Tracey McCulloch, Eric S. Schaefer

**Affiliations:** 1 Department of Biomedical Sciences, Humanitas University, Milan, Italy; 2 Department of Oncology and Hematology, IRCCS Humanitas Research Hospital, Humanitas Cancer Center, Via Manzoni 56, Rozzano-Milan, 20089 Italy; 3 Department of Medical Oncology, Hospital Ramón Y Cajal, Madrid, Spain; 4 Department of Medical Oncology, National Cancer Center Singapore, Singapore, Singapore; 5 Department of Medical Oncology, University Hospital 12 de Octubre, Madrid, Spain; 6 Department of Medical Oncology and Hematology, Princess Margaret Cancer Centre, Toronto, ON Canada; 7 Department of Medical Oncology, Centro di Riferimento Oncologico - CRO, Aviano, Italy; 8 Department of Multidisciplinary Oncology and Therapeutic Innovations, Aix Marseille University, CNRS, INSERM, CRCM, APHM, CEPCM, CLIP, Marseille, France; 9 Faculté de Médecine, Université Paris Saclay, Kremlin Bicêtre, France; 10 Medical Oncology Department, Gustave Roussy, Villejuif, France; 11 Department of Internal Medicine, Seoul National University Hospital, Seoul, South Korea; 12 Department of Hematology and Medical Oncology, University Medical Center Göttingen, Göttingen, Germany; 13 Department of Medical Oncology Service, Vall d’Hebron University Hospital and Vall d’Hebron Institute of Oncology, Barcelona, Spain; 14 Novartis Pharmaceuticals Corporation, East Hanover, NJ USA; 15 Novartis Pharma AG, Basel, Switzerland; 16 Department of Medical Oncology, Highlands Oncology Group, Fayetteville, AZ USA

**Keywords:** Canakinumab, NSCLC, PD-L1, Platinum-doublet chemotherapy, Spartalizumab

## Abstract

**Background:**

Despite promising outcomes of treatment with anti-programmed cell death (PD)-1/PD-ligand (L)1 agents in combination with platinum-doublet chemotherapy (PDC) in the first-line setting, a significant unmet medical need remains in patients with PD-L1-unselected non-small cell lung cancer (NSCLC).

**Methods:**

This multicenter, open-label, phase 1b study comprising dose-confirmation and dose-expansion parts investigated the combination of spartalizumab and various PDC regimens, with or without canakinumab, in treatment-naïve patients with PD-L1-unselected, metastatic NSCLC. The primary objectives were to determine maximum tolerated dose (MTD) and/or recommended dose for expansion (RDE) of spartalizumab, with or without canakinumab, in combination with PDC in the dose-confirmation part and antitumor activity of spartalizumab in the dose-expansion part.

**Results:**

The MTD/RDE of spartalizumab was 300 mg every 3 weeks (Q3W) when administered with either gemcitabine (1250 mg/m^2^)/cisplatin (75 mg/m^2^) (group A; no dose-limiting toxicities [DLTs]), pemetrexed (500 mg/m^2^)/cisplatin (group B; 2 DLTs: grade 2 posterior reversible encephalopathy syndrome and grade 4 hyponatremia), or paclitaxel (200 mg/m^2^)/carboplatin area under the curve 6 min*mg/mL (group C; 1 DLT: grade 4 neutropenic colitis). The RDE of canakinumab combined with spartalizumab and pemetrexed/cisplatin (group E; no DLTs) was 200 mg Q3W (no dose-expansion part was initiated). No new safety signals were identified. In groups A, B, C, and E, the overall response rates were 57.6%, 55.3%, 51.5%, and 57.1%, respectively. Group B compared with other groups had the longest median progression-free survival (10.4 months vs. 6.2–7.5 months), overall survival (29.7 months vs. 16.1–21.0 months), and duration of response (30.1 months vs. 6.0-8.2 months).

**Conclusions:**

The combination of spartalizumab and PDC, with or without canakinumab, was well tolerated across treatment groups. The antitumor activity across treatment groups was comparable with that of pembrolizumab and pemetrexed combination. Canakinumab did not appear to improve the antitumor activity when combined with spartalizumab, pemetrexed and cisplatin.

**Trial registration:**

The trial was registered in Clinicaltrials.gov with identifier no. NCT03064854. Date of Registration: 06 February 2017.

**Supplementary Information:**

The online version contains supplementary material available at 10.1186/s12885-024-12841-2.

## Highlights

A significant unmet medical need still exists in patients with PD-L1-unselected NSCLC due to the limited efficacy of immune checkpoint inhibitors and the lack of a well-defined, effective treatment approach.

Spartalizumab in combination with platinum doublet chemotherapy (PDC), with/without canakinumab, was well tolerated across treatment groups.

Spartalizumab plus PDC, with/without canakinumab, showed the antitumor activity in this clinical setting.

Addition of canakinumab did not appear to improve the antitumor activity of the combination of spartalizumab with pemetrexed plus cisplatin.

**Supplementary Information:**

The online version contains supplementary material available at 10.1186/s12885-024-12841-2.

## Background

Non-small cell lung cancer (NSCLC) accounts for approximately 85% of all lung cancer cases [1]. The average 5-year survival rate for all patients with lung cancer after diagnosis is 21%, which decreases to 6% for patients with metastatic disease at diagnosis [2].Platinum-doublet chemotherapy (PDC) (cisplatin [CIS] or carboplatin [CARBO] combined with another chemotherapy agent, with or without bevacizumab) was historically the standard first-line treatment for patients with locally advanced/metastatic NSCLC [3, 4].Platinum-pemetrexed (PEM) chemotherapy emerged as a basis for effective first-line treatment for metastatic NSCLC with non-squamous histology, whereas CIS or CARBO combined with either gemcitabine (GEM), paclitaxel (PAC), docetaxel, or vinorelbine were recommended for treatment of patients with squamous NSCLC [5–10]. However, overall survival (OS) remains limited to a subset of patients with locally advanced/metastatic NSCLC treated with the first-line chemotherapy [6, 8, 11–13].

Numerous studies have shown that blockade of the programmed cell death protein-1 (PD-1) pathway promotes robust antitumor response in patients with different tumor types [13–16]. In patients with NSCLC, pembrolizumab, an anti–PD-1 therapy, in the first-line setting showed superior efficacy compared with PDC in those patients who had PD-L1 expression in > = 50% of viable tumor cells without epidermal growth factor receptor (EGFR)/anaplastic lymphoma kinase (ALK) aberrations [17, 18]. The anti–PD-(L)1 agents (pembrolizumab, nivolumab, and atezolizumab) alone or combined with PDC in the first-line setting showed prolonged progression-free survival (PFS) and OS and better overall response rate (ORR) versus chemotherapy alone and acceptable safety profiles [18–21]. Thus, first-line regimens comprising immunotherapy and chemotherapy are now recommended for the treatment of patients with NSCLC in the National Comprehensive Cancer Network guidelines [22, 23].

Spartalizumab (SPARTA) is a high-affinity, humanized immunoglobulin 4κ antibody that binds PD-1 and blocks the interaction with its ligands PD-L1 and PD-L2. In a phase 1 study, SPARTA was well tolerated at all doses tested in previously treated patients with advanced solid tumors and resulted in immune activation in tumors [15].

Despite promising treatment outcomes, a significant unmet medical need in patients with PD-L1-unselected NSCLC remains [24]. Immune checkpoint inhibitors, like anti-PD-1 and anti-PD-L1, show long-term benefits in NSCLC, but their efficacy is less certain for PD-L1-negative patients. Combining anti-PD-1 or anti-PD-L1 with platinum chemotherapy improves survival over chemotherapy alone, but specific benefits for PD-L1-negative patients are unclear. Large trials show promising results for high PD-L1 expression, but not for PD-L1-negative patients, highlighting the need for more targeted therapies [25]. Owing to the complexity of the immune-tumor interactions, the addition of investigational immuno-oncology agents having a complimentary mechanism of action may further improve clinical outcomes. Canakinumab (CAN) is an investigational, high-affinity, human monoclonal antibody that specifically binds to interleukin (IL)-1β [26]. This leads to suppression of tumor progression and enhancement of antitumor immunity through maturation of myeloid-derived suppressor cells into M1 (antitumor) macrophages within the tumor microenvironment [27, 28]. CAN was shown to inhibit the inflammatory signaling conveyed by the IL-1β-C-reactive protein axis and target immunosuppressive cells in the tumor microenvironment [28, 29].

Here, we report the results of a phase 1b study that investigated the combination of SPARTA, with or without CAN, and various PDC regimens to determine the recommended doses and regimens for expansion and further evaluation of the safety and efficacy of these combinations in advanced NSCLC with PD-L1-unselected with squamous and non-squamous histology.

## Methods

### Study design and patient population

This was a multicenter, open-label, phase 1b study (NCT03064854) [30] investigating PDC regimens in combination with SPARTA, with or without CAN, in treatment-naïve adult patients with histologically or cytologically confirmed stage IIIB-C/IV or relapsed, locally advanced, or metastatic squamous/non-squamous NSCLC lacking *EGFR*-sensitizing mutations and/or *ALK-* or *ROS1* rearrangements. Enrolled patients had an Eastern Cooperative Oncology Group performance status (ECOG PS) of 0–1 and ≥ 1 measurable tumor lesion as per Response Evaluation Criteria in Solid Tumors version 1.1 (RECIST v1.1). Patients with any history of interstitial lung disease, interstitial pneumonitis, or leptomeningeal metastases, and/or those who had received thoracic radiotherapy to lung fields ≤ 4 weeks prior to the study treatment, had not recovered from radiotherapy-related toxicities or had any other malignancy not treated in this study were excluded from the study. Patients treated with CAN or other immune-targeting agents prior to this study were not included in group E. PD-L1 expression was assessed but not used to determine eligibility.

The study comprised both dose-confirmation and dose-expansion parts (Supplementary Figure S1). The dose-confirmation part determined the maximum tolerated dose (MTD)/recommended dose for expansion (RDE) of SPARTA/CAN in combination with 3 unique PDC regimens, based on the dose-limiting toxicities (DLTs) observed using a Bayesian logistic regression model (BLRM). The dose-confirmation part had 4 groups (A, B, C and E) with up to four treatment cycles for each group. In group A, patients with squamous NSCLC were treated with GEM (1250 mg/m^2^)/CIS (75 mg/m^2^) and SPARTA (initial dose level 300 mg intravenous [i.v.] every 3 weeks [Q3W] and dose level-1 [DL1] at 300 mg i.v. Q6W). In group B, patients with non-squamous NSCLC were treated with PEM (500 mg/m^2^)/CIS (75 mg/m^2^) and SPARTA. In group C, patients with squamous or non-squamous NSCLC were treated with PAC (200 mg/m^2^)/CARBO (target area under the curve [AUC] 6 min*mg/mL) and SPARTA. Group D was planned to include patients with non-squamous NSCLC who were to receive PDC with or without SPARTA in the second-line setting. Recruitment of patients to group D was not initiated and it was removed during the second protocol amendment in order to add group E. In group E, patients with non-squamous NSCLC were treated with PEM (500 mg/m^2^)/CIS (75 mg/m^2^), SPARTA, and CAN (initial dose at 200 mg Q3W subcutaneous [s.c.] and DL1 at 200 mg s.c. Q6W). Dose modifications for PDC were as per locally approved product labels. No dose reduction was allowed for SPARTA in any group or for CAN in group E.

The dose-expansion part was initiated once the MTD and/or RDE were established for groups A, B, and C. The dose-expansion part for group E was not initiated and recruitment to this group was halted after careful evaluation of the competitive therapeutic landscape for lung cancer and the slow recruitment to the dose-confirmation part for group E. The recruitment halt was not due to any safety concerns.

### Study objectives

The primary objective in the dose confirmation part was to establish the MTD/RDE of SPARTA in combination with PDC in groups A, B, and C, and the MTD/RDE of CAN in combination with SPARTA and PDC in group E based on the incidence of DLTs in the first 6 weeks of therapy. Primary objective in the dose-expansion part was to assess antitumor activity of SPARTA in combination with PDC as measured by overall response rate (ORR) per RECIST v1.1 in groups A, B, and C.

For groups A, B, and C, secondary objectives were the assessment of antitumor activity (measured by PFS, disease control rate [DCR], duration of response [DOR], and time to response [TTR]), OS, safety/tolerability, pharmacokinetics (PK), and the prevalence and incidence of immunogenicity of SPARTA in combination with PDC. For group E, secondary objectives were the assessment of ORR, PFS, DCR, DOR, TTR, OS, safety/tolerability, PK, and the prevalence and incidence of immunogenicity of CAN in combination with SPARTA and PDC.

Exploratory objectives included assessment of antitumor activity based on immune-related response criteria (irRC), association of PD-L1 expression in tumor tissue with clinical activity using immunohistochemistry on NSCLC samples and detected through monoclonal mouse anti-PD-L1, Clone 22C3. Additionally, the effects of chemotherapy combinations on the PK profile of SPARTA or vice versa, were examined.

### Statistical methods

All analyses were performed by Novartis Pharmaceuticals. OS and TTR were estimated with the Kaplan-Meier method. SAS version 9.4 or later software was used to perform all data analyses and to generate data outputs. No hypothesis was tested. The dose was confirmed by the BLRM. The efficacy analysis was performed on the full-analysis set, which comprised patients who received at least 1 dose of study drug. ORR was estimated and the exact binomial 95% CI was reported by each group. PFS and DOR were assessed using Kaplan-Meier method.

## Results

### Baseline characteristics

On the data cut-off date (July 29, 2021), 112 of 156 patients completed screening and 111 patients were enrolled in the dose-confirmation and dose-expansion parts of the study. One patient was not treated due to physician’s decision. The primary reason for treatment discontinuation was disease progression (Supplementary Figure S2).

Baseline demographics and disease characteristics are presented in Table 1. Overall, baseline characteristics across the treatment groups were well balanced. The median age was comparable, except for group A, where a higher proportion of patients were aged ≥ 65 years (51.5%) compared with other groups (Table 1). Males were predominant in groups A (81.8%) and E (85.7%). All evaluable patients had an ECOG PS of 0–1. Most of the patients in all groups were Caucasian (68.4-84.8%) and had stage IV disease (69.7-86.8%) at initial diagnosis. Patients in group A had squamous NSCLC, whilst patients in groups B and E had non-squamous NSCLC, and patients in group C had squamous or non-squamous NSCLC. Prior radiotherapy across all groups was received by 12.1-36.4% patients, and 5.3-28.6% of patients had received prior surgery.

**Table 1:** Baseline demographics and disease characteristics (full analysis set)

Demographic variable	Group A (SPARTA/GEM/CIS) *N* = 33	Group B (SPARTA/PEM/CIS) *N* = 38	Group C (SPARTA/CARBO/PAC) *N* = 33	Group E (SPARTA/CAN/PEM/CIS) *N* = 7
Median age, years (IQR)	65.0 (57.0–68.0)	63.0 (58.0–69.0)	60.0 (55.0–68.0)	62.0 (57.0–72.0)
Age category (years), n (%)				
18 to < 65	16 (48.5)	22 (57.9)	21 (63.6)	4 (57.1)
≥ 65	17 (51.5)	16 (42.1)	12 (36.4)	3 (42.9)
Sex, n (%)				
Female	6 (18.2)	20 (52.6)	19 (57.6)	1 (14.3)
Male	27 (81.8)	18 (47.4)	14 (42.4)	6 (85.7)
ECOG PS, n (%)				
0	8 (24.2)	13 (34.2)	10 (30.3)	1 (14.3)
1	25 (75.8)	24 (63.2)	23 (69.7)	6 (85.7)
Missing	0	1 (2.6)	0	0
Race, n (%)				
Caucasian	26 (78.8)	26 (68.4)	28 (84.8)	5 (71.4)
Black	0	2 (5.3)	0	0
Asian	5 (15.2)	6 (15.8)	3 (9.1)	2 (28.6)
Native American	0	0	1 (3.0)	0
Other/Unknown	2 (6.0)	4 (10.5)	1 (3.0)	0
Histological subtype, n (%)				
Adenocarcinoma	0	38 (100)	22 (66.7)	7 (100)
Squamous cell carcinoma	33 (100)	0	8 (24.2)	0
Other	0	0	3 (9.1)	0
Stage at initial diagnosis, n (%)				
III	3 (9.1)	1 (2.6)	3 (9.1)	1 (14.3)
IV	28 (84.8)	33 (86.8)	23 (69.7)	6 (85.7)
Metastatic sites at baseline, n (%)				
Lung	21 (63.6)	28 (73.7)	22 (66.7)	5 (71.4)
Lymph node	19 (57.6)	27 (71.1)	26 (78.8)	3 (42.9)
Liver	12 (36.4)	4 (10.5)	10 (30.3)	1 (14.3)
Bone	7 (21.2)	14 (36.8)	14 (42.4)	3 (42.9)
Pleura	5 (15.2)	8 (21.1)	7 (21.2)	3 (42.9)
Brain	2 (6.1)	6 (15.8)	9 (27.3)	0
Prior therapy, n (%)				
Any therapy	28 (84.8)	31 (81.6)	27 (81.8)	7 (100)
Radiotherapy	4 (12.1)	10 (26.3)	12 (36.4)	2 (28.6)
Surgery (excluding biopsy)	2 (6.1)	2 (5.3)	4 (12.1)	2 (28.6)

CAN, canakinumab; CARBO, carboplatin; CIS, cisplatin; ECOG PS, Eastern Cooperative Oncology Group performance status; GEM, gemcitabine; IQR, interquartile range; PAC, paclitaxel; PEM, pemetrexed; SPARTA, spartalizumab

Median duration of exposure was 33.4 (interquartile range [IQR]: 21.0−84.0), 44.4 (IQR: 18.0−86.9), 27.0 (IQR: 18.0−45.0) and 33.0 (IQR: 24.0−53.9) weeks in groups A, B, C, and E, respectively. In most of the patients, the dose reductions or interruptions were due to adverse events (AEs) irrespective of treatment regimen.

### Determination of MTD/RDE

Overall, three patients reported DLTs during the first two cycles (6 weeks): two patients in group B (grade 2 posterior reversible encephalopathy syndrome in one patient and grade 4 hyponatremia in another patient) and one patient in group C (grade 4 neutropenic colitis). No DLTs were reported in groups A and E. Based on the BLRM model and DLTs, the MTD/RDE of SPARTA was declared at a dose of 300 mg when administered Q3W either in combination with GEM 1250 mg/m^2^ and CIS 75 mg/m^2^ for 4 cycles followed by maintenance with SPARTA in group A; or with CIS 75 mg/m^2^ and PEM 500 mg/m^2^ for 4 cycles followed by maintenance with SPARTA and PEM in group B; or with PAC 200 mg/m^2^ and CARBO AUC 6 min*mg/mL for 4 cycles followed by maintenance with SPARTA in group C. In group E, CAN at a dose of 200 mg Q3W in combination with SPARTA 300 mg Q3W, PEM 500 mg/m^2^, and CIS 75 mg/m^2^ for 4 cycles followed by maintenance with the combination of SPARTA, CAN, and PEM was proposed as the RDE, based on the BLRM model and the safety data.

### Safety

All patients reported at least one AE of any grade regardless of the study drug relationship. All patients in groups B and E and 97% patients in groups A and C had ≥ 1 AE suspected to be related to the study drug (Supplementary Table S1; Table 2).

**Table 2:** Overview of safety data (safety analysis set)

	Group A (SPARTA/GEM/CIS) *N* = 33 *n* (%)	Group B (SPARTA/PEM/CIS) *N* = 38 *n* (%)	Group C (SPARTA/CARBO/PAC) *N* = 33 *n* (%)	Group E (SPARTA/CAN/PEM/CIS) *N* = 7 *n* (%)
AEs	33 (100.0)	38 (100.0)	33 (100.0)	7 (100.0)
Treatment-related AEs	32 (97.0)	38 (100.0)	32 (97.0)	7 (100.0)
Grade ≥ 3 AEs	29 (87.9)	31 (81.6)	27 (81.8)	5 (71.4)
Treatment-related ≥ 3 AEs	20 (60.6)	23 (60.5)	21 (63.6)	3 (42.9)
SAEs	15 (45.5)	22 (57.9)	13 (39.4)	4 (57.1)
Treatment-related SAEs	4 (12.1)	14 (36.8)	4 (12.1)	0
Fatal SAEs	2 (6.1)	3 (7.9)	0	1 (14.3)
Treatment-related fatal SAEs	0	1 (2.6)	0	0
AEs leading to discontinuation	4 (12.1)	13 (34.2)	3 (9.1)	3 (42.9)
Treatment-related AEs leading to discontinuation	2 (6.1)	11 (28.9)	2 (6.1)	2 (28.6)
AEs leading to dose adjustment/interruption	26 (78.8)	22 (57.9)	20 (60.6)	5 (71.4)
AEs requiring additional therapy	33 (100.0)	37 (97.4)	32 (97.0)	7 (100.0)

AE, adverse event; CAN, canakinumab; CARBO, carboplatin; CIS, cisplatin; GEM, gemcitabine; PAC, paclitaxel; PEM, pemetrexed; SAE, serious AE; SPARTA, spartalizumab

#### Group A (Squamous/SPARTA-GEM-CIS)

The most frequent treatment-related AEs (TRAEs) of any grade were anemia, neutropenia, asthenia and nausea, and the most frequent grade ≥ 3 TRAEs were neutropenia (27.3%) and thrombocytopenia (15.2%) (Supplementary Table S1). Fifteen patients (45.5%) reported serious AEs (SAEs), and treatment-related SAEs were reported in 4 patients (12.1%) (Supplementary Tables S2, S3; Table 2). Three on-treatment deaths were reported. The reason of death was NSCLC (1 patient), myocardial infarction (1 patient) and septic shock (1 patient).

#### Group B (Non-squamous/SPARTA-PEM-CIS)

The most common TRAEs of any grade were nausea, neutropenia anemia and vomiting, and grade ≥ 3 TRAEs were neutropenia (34.2%), anemia and leukopenia (10.5% each) (Supplementary Table S1). Twenty-two patients (57.9%) reported SAEs, and treatment-related SAEs were reported in 14 patients (36.8%) (Supplementary Tables S2, S3; Table 2). Three on-treatment deaths were reported. The reason of death was cardiac arrest (2 patient) and sepsis (1 patient).

#### Group C ([Non-]squamous/SPARTA-CARBO-PAC)

The most common TRAEs of any grade were neutropenia, anemia, asthenia, nausea, and decreased appetite, and grade ≥ 3 treatment-related AEs were neutropenia (45.5%) and thrombocytopenia (12.1%) (Supplementary Table S1). Thirteen patients (39.4%) reported SAEs; the SAE of general physical health deterioration resulted in death in one patient. Treatment-related SAEs reported in four patients (12.1%) (Supplementary Tables S2, S3; Table 2). One on-treatment death was reported due to study indication.

#### Group E (Non-squamous/SPARTA-CAN-PEM-CIS)

The most common TRAEs of any grade were fatigue, decreased appetite, anemia and blood creatinine increased, and grade ≥ 3 TRAEs were anemia (28.6%) and neutropenia (14.3%) (Supplementary Table S1). No AE led to treatment discontinuation. Four patients (57.1%) reported SAEs. No SAE was suspected to be treatment related. (Supplementary Tables S2, S3; Table 2). The SAE of pneumonia resulted in death in one patient.

In 58–79% of 22 patients, AEs led to dose adjustment or interruption (Supplementary Table S4), and in 9-34.2% patients, AEs led to treatment discontinuation in groups A, B and C (Supplementary Table S5). The AEs irrespective of treatment regimen requiring additional medication or therapies were reported in almost all (97–100%) patients (Supplementary Table S6).

The majority of AEs of special interest (AESIs) related to SPARTA were of grade 1 or 2. Grade ≥ 3 AESIs related to SPARTA were colitis/diarrhea in groups B (*n* = 4), C (*n* = 3), and E (*n* = 1); rash (*n* = 1) and nephritis (*n* = 4) in group B; and type 1 diabetes mellitus (*n* = 1) in group E (Supplementary Table S7). The most frequent AESI irrespective of study drug relationship in group E was infection reported in five patients (71.4%). No grade ≥ 3 AESIs was suspected to be treatment related.

### Efficacy

#### Group A (Squamous/SPARTA-GEM-CIS)

**Fig. 1:**
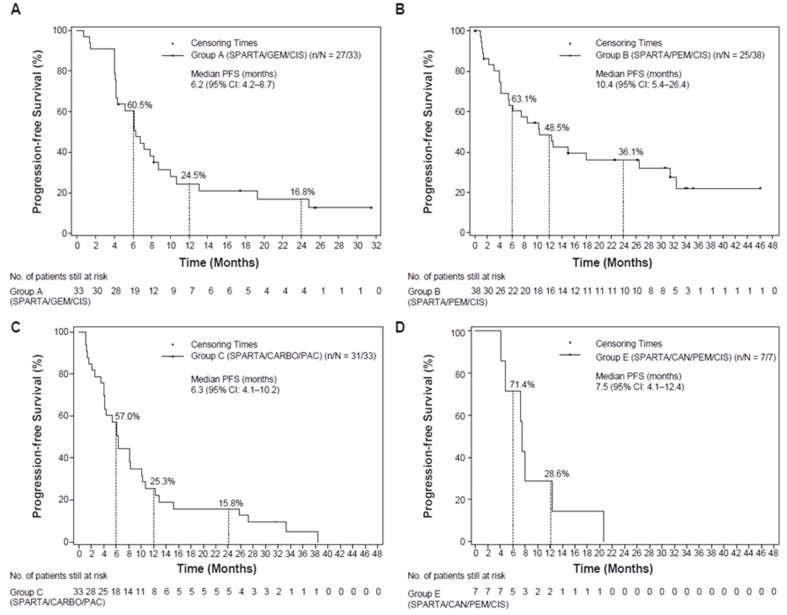
Kaplan-Meier plots of the median PFS based on investigator’s assessment in group A (**A**), group B (**B**), group C (**C**) and group E (**D**). CAN, canakinumab; CARBO, carboplatin; CI, confidence interval; CIS, cisplatin; GEM, gemcitabine; PAC, paclitaxel; PEM, pemetrexed; PFS, progression-free survival; SPARTA, spartalizumab

**Fig. 2:**
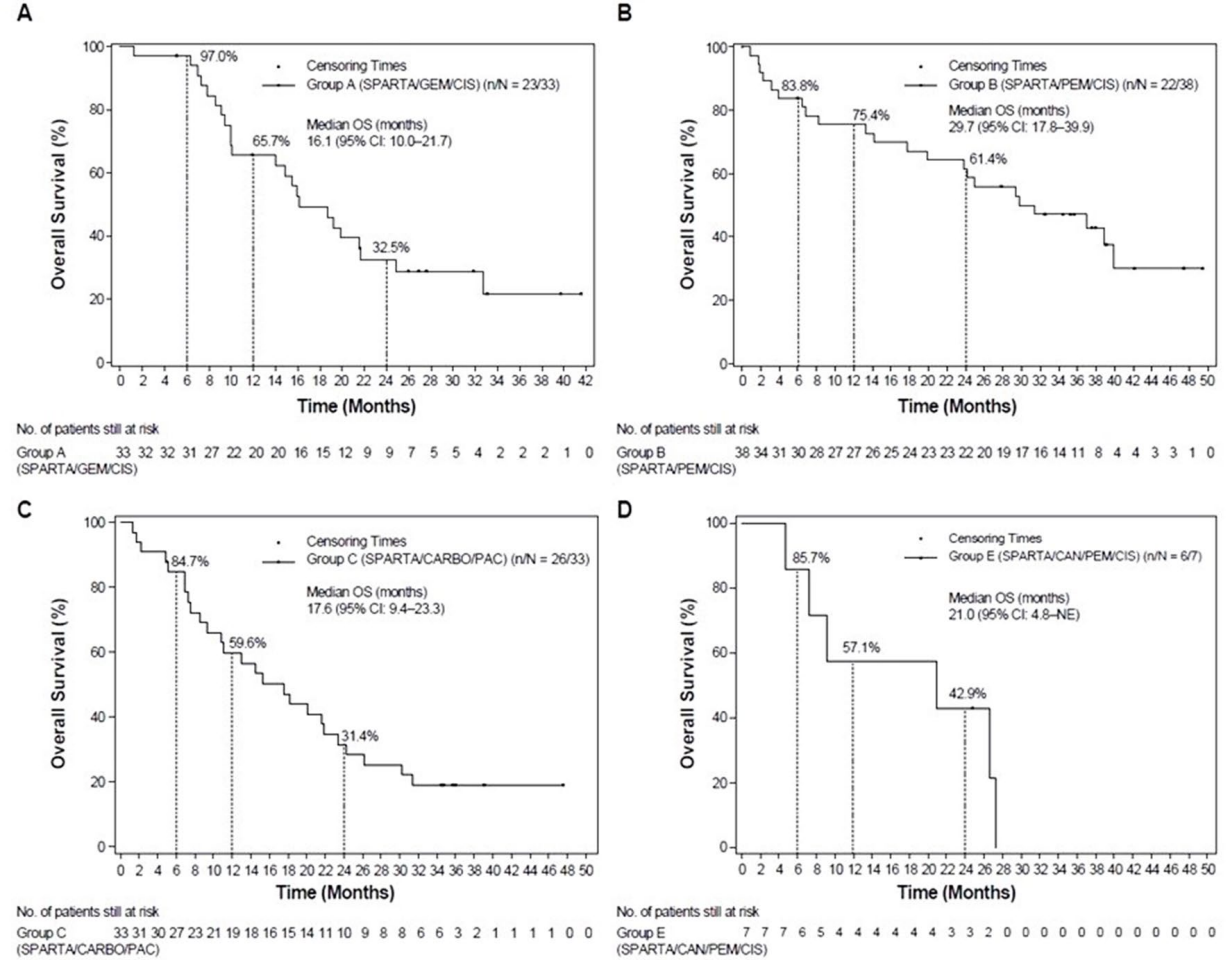
Kaplan-Meier plots of the median OS based on investigator’s assessment in group A (**A**), group B (**B**), group C (**C**) and group E (**D**). CAN, canakinumab; CARBO, carboplatin; CI, confidence interval; CIS, cisplatin; GEM, gemcitabine; OS, overall survival; PAC, paclitaxel; PEM, pemetrexed; SPARTA, spartalizumab

ORR as assessed by investigator was 57.6% (95% CI: 39.2–74.5), with a complete response (CR) in one patient and partial response (PR) in 18 patients (Table 3). The DCR (CR + PR + SD) as per investigator’s assessment was 90.9% (95% CI: 75.7–98.1). The median PFS, OS, DOR, and TTR were 6.2 months (95% CI: 4.2–8.7), 16.1 months (95% CI: 10.0-21.7), 6.0 months (95% CI: 3.0–18.0), and 2.7 months (95% CI: 1.3-not estimable [NE]), respectively (Supplementary Table S8; Figs. 1 and 2).

**Table 3:** BOR, ORR and DCR based on investigator’s assessment

	Group A (SPARTA/GEM/CIS) *N* = 33	Group B (SPARTA/PEM/CIS) *N* = 38	Group C (SPARTA/CARBO/PAC) *N* = 33	Group E (SPARTA/CAN/PEM/CIS) *N* = 7
Best overall response, n (%)				
CR	1 (3.0)	2 (5.3)	2 (6.1)	0
PR	18 (54.5)	19 (50.0)	15 (45.5)	4 (57.1)
SD	11 (33.3)	10 (26.3)	10 (30.3)	3 (42.9)
PD	3 (9.1)	4 (10.5)	4 (12.1)	0
Unknown	0	3 (7.9)	2 (6.1)	0
ORR: CR + PR, n (%; 95% CI)	19 (57.6; 39.2–74.5)	21 (55.3; 38.3–71.4)	17 (51.5; 33.5–69.2)	4 (57.1; 18.4–90.1)
DCR: CR + PR + SD, n (%; 95% CI)	30 (90.9; 75.7–98.1)	31 (81.6; 65.7–92.3)	27 (81.8; 64.5–93.0)	7 (100; 59.0-100)

BOR, best overall response; CAN, canakinumab; CARBO, carboplatin; CI, confidence interval; CIS, cisplatin; CR, complete response; DCR, disease control rate; GEM, gemcitabine; ORR, overall response rate; PAC, paclitaxel; PEM, pemetrexed; PD, progressive disease; PR, partial response; SD, stable disease; SPARTA, spartalizumab

#### Group B (Non-squamous/SPARTA-PEM-CIS)

The ORR was 55.3% (95% CI: 38.3–71.4), with two patients reporting a CR and 19 patients having a PR (Table 3). The DCR was 81.6% (95% CI: 65.7–92.3). The median PFS, OS, DOR, and TTR were 10.4 months (95% CI: 5.4–26.4), 29.7 months (95% CI: 17.8–39.9), 30.1 months (95% CI: 9.0−NE), and 6.2 months (95% CI: 1.4-NE), respectively (Supplementary Table S8; Figs. 1 and 2).

#### Group C([Non-]squamous/SPARTA-CARBO-PAC)

The ORR was 51.5% (95% CI: 33.5–69.2), with a CR reported in two patients and a PR reported in 15 patients (Table 3). The DCR was 81.8% (95% CI: 64.5–93.0). The median PFS, OS, DOR, and TTR were 6.3 months (95% CI: 4.1–10.2), 17.6 months (95% CI: 9.4–23.3), 8.2 months (95% CI: 5.1–23.1) and 3.9 months (95% CI: 1.3-NE), respectively (Supplementary Table S8; Figs. 1 and 2).

Patients with nonsquamous histology had greater ORR than those with squamous histology (63.6%; 95% CI: 40.7–82.8 vs. 37.5%; 95% CI: 8.5–75.5). Likewise, the DCR was higher in patients with non-squamous histology (90.9%; 95% CI: 70.8–98.9) than in patients with squamous histology (75.0%; 95 CI: 34.9–96.8). The antitumor activity assessed by ORR, PFS, DCR, DOR, and TTR by irRC was comparable with that assessed by RECIST v1.1 (data not shown).

#### Group E (Non-squamous/SPARTA-CAN-PEM-CIS)

The ORR was 57.1% (95% CI: 18.4–90.1), with four patients reporting a PR. No patients had a CR in group E (Table 3). The DCR was 100% (95% CI: 59.0-100). The median PFS, OS, DOR and median TTR were 7.5 months (95% CI: 4.1–12.4), 21.0 months (95% CI: 4.8-NE), 7.1 months (95% CI: 1.4-NE) and 5.0 months (95% CI: 1.3-NE), respectively (Supplementary Table S8; Figs. 1 and 2).

### Association of tumor PD-L1 expression levels with antitumor response

Tumor proportion score was assessed for all patients with PD-L1 expression of < 1%, ≥ 1% to < 50%, and ≥ 50% and evaluated for their association with ORR, PFS, and DCR. A trend towards an increased proportion of clinical response in the PD-L1 TPS ≥ 50% subgroup compared with the PD-L1 < 1% and the PD-L1 ≥ 1% to < 50% subgroups was evident in groups B, C, and E (Supplementary Table S9). The ORR was higher in the PD-L1 ≥ 50% subgroup than in the PD-L1 < 1% and the ≥ 1% to < 50% subgroups in groups B, C, and E, except in group A, where the ORR was higher in the PD-L1 ≥ 1% to < 50% subgroup than in the < 1% and the ≥ 50% subgroups. The median PFS was also higher in the PD-L1 ≥ 50% subgroup than in the PD-L1 < 1% and the PD-L1 ≥ 1% to < 50% subgroups in all groups, except group A, where the median PFS was similar in the PD-L1 < 1% and the PD-L1 ≥ 1% to < 50% subgroups. The median PFS was NE in the subgroup with PD-L1 TPS of ≥ 50%.

In groups A and B, the DCR was higher in the subgroup with PD-L1 TPS of ≥ 50% compared with the PD-L1 < 1% and the PD-L1 ≥ 1% to < 50% subgroups; whilst in group C, the DCR was higher in the PD-L1 < 1% subgroup compared with the PD-L1 ≥ 1% to < 50% and ≥ 50% subgroups. In group E, the DCR was similar (100%) across all PD-L1 TPS subgroups. Due to limitation of post-baseline PD-L1 data, absolute or relative change in PD-L1 expression from baseline and by visit could not be analyzed.

### Pharmacokinetics (PK) and immunogenicity

Following administration of the combination of SPARTA with CAN and chemotherapy agents (PAC, PEM, GEM, and CIS), the PK parameters of each compound were comparable across groups (Supplementary Table S10). There was no clear difference between PK parameters in groups B and E, indicating that CAN had no effect on the PK of SPARTA. In group A, the C_trough_ (geo-CV%) on day 1 of cycle 1 (*n* = 24), cycle 3 (*n* = 22) and cycle 4 (*n* = 22) was 15.6 µg/mL (27.2%), 37.2 µg/mL (29.8%) and 42.2 µg/mL (31.3%), respectively. In group B, the C_trough_ on day 1 of cycle 1 (*n* = 29), cycle 3 (*n* = 25) and cycle 4 (*n* = 24) was 18.4 µg/mL (34.6%), 34.8 µg/mL (52.6%) and 50.8 µg/mL (28.4%), respectively. In group C, the C_trough_ on day 1 of cycle 1 (*n* = 30), cycle 3 (*n* = 18) and cycle 4 (*n* = 19) was 15.4 µg/mL (71.7%), 35.4 µg/mL (39.0%) and 39.7 µg/mL (41.5%), respectively. In group E, the C_trough_ on day 1 of cycle 1 (*n* = 7), cycle 3 (*n* = 5) and cycle 4 (*n* = 6) was 19.9 µg/mL (36.3%), 46.7 µg/mL (17.8%) and 46.1 µg/mL (41.1%), respectively (Supplementary Table S11).

The incidence of SPARTA immunogenicity was 3.4% and 9.4% in groups A and B, respectively, and no CAN immunogenicity was detected (data not shown).

## Discussion

This phase 1b study evaluated the safety and tolerability of SPARTA in combination with different PDC treatments and identified the RDE for the dose-expansion part as well as determined the MTD of CAN in combination with SPARTA and PDC in patients with NSCLC. Additionally, the preliminary antitumor activity and its association with the PD-L1 expression levels in tumors as well as PK profiles were investigated for each treatment group [30].

Chemotherapy may reduce “off target” immunosuppression in the tumor microenvironment while also increasing antigenicity through the immunogenic cell death of tumor cells [31]. The combination of chemotherapy and PD-1 blocking therapy has yielded positive outcomes in the early treatment of NSCLC by harnessing the potential synergy between both drugs [32].

SPARTA in combination with CAN and PAC, PEM, GEM, and CIS was well tolerated across the treatment groups. The safety profile of SPARTA appeared to be consistent with that reported in previous studies [24, 33]. The SPARTA in combination with PDC, with or without CAN, was safe, and the reported AEs were manageable with dose adjustments/interruptions and/or additional medications or therapies according to the AE management guidelines predefined in the protocol. Both grade 3/4 treatment-related AEs and SAEs suspected to be related to the treatment were consistent with the known safety profile of SPARTA. No treatment-related deaths were reported. Two of the 8 on-treatment deaths were attributed to the study indication (NSCLC) and a single case of sepsis was suspected to be related to other study treatment (non-investigational). The AE of infection with CAN treatment appeared to be consistent with that reported in the pooled group of patients with CAN vs. placebo in the CANTOS trial. Therefore, patients treated with CAN should be carefully monitored for early signs and symptoms of serious infection similarly when using other biologic immunomodulators [34].

SPARTA in combination with different PDC regimens demonstrated antitumor activity and favorable OS. The previous studies have reported the improvement in ORR and DOR with the combination of pembrolizumab and PEM/platinum as first-line therapy in patients with metastatic non-squamous NSCLC, regardless of the PD-L1 expression levels [28, 35, 36]. In a few studies, the benefit of nivolumab combined with platinum-based therapy in the first-line setting in improving the ORR and prolonging PFS, and OS was limited [37, 38]. However, addition of ipilimumab (anti-CTLA4) to nivolumab with or without chemotherapy provided a significant OS benefit along with a favorable risk-benefit profile [38, 39,40]. Compared with other combinations of SPARTA and PDC (GEM/CIS and PAC/CARBO), group B had the longest median PFS, DOR, and OS. The addition of CAN to the combination of SPARTA and PEM/CIS did not appear to improve the antitumor activity of this regimen. However, due to the small sample size, these results should be interpreted with caution. The direct comparison of treatment groups could not be performed due to different histological subtypes and chemotherapy regimens.

A trend towards greater antitumor activity in the PD-L1 TPS of ≥ 50% subgroups compared with the PD-L1 < 50% subgroups in all treatment groups was observed, except a few instances. In previous reports also, a high PD-L1 expression was correlated with a significantly higher DCR and longer PFS in NSCLC patients treated with nivolumab or pembrolizumab [18, 41]. However, these results should be interpreted with caution due to the small number of patients in the PD-L1 ≥ 50% subgroup.

The PK parameters of SPARTA in combination with CAN and chemotherapy agents were comparable across combination groups and generally comparable to those observed in previous studies, with the exception of the combination with CIS and CARBO [42–45], which differed slightly from previously reported values that may be related to the limitations of the sampling schedule [36]. It is unlikely that SPARTA affects the clearance and exposure of either compound by the virtue of elimination of chemotherapy agents (renal clearance and irreversible protein binding) [46]. The addition of CAN to the SPARTA, PEM, and CIS regimen did not affect the PK parameters of SPARTA. Overall, the data support a low likelihood of drug-drug interactions between the study drugs.

## Conclusion

This study showed that SPARTA in combination with PDC, with or without CAN, was well tolerated across all treatment groups. No new safety concerns were identified, and most of the AEs were manageable. Overall, the safety profile was in line with that of known SPARTA safety profile and mostly in line with the expected toxicity by chemotherapy and the combination showed the antitumor activity in this clinical setting.

## Electronic supplementary material

Below is the link to the electronic supplementary material.

Supplementary Material 1

## Data Availability

Novartis will not provide access to patient-level data, if there is a reasonable likelihood that individual patients could be re-identified. Phase 1 studies, by their nature, present a high risk of patient re-identification; therefore, patient individual results for phase 1 studies cannot be shared. In addition, clinical data, in some cases, have been collected subject to contractual or consent provisions that prohibit transfer to third parties. Such restrictions may preclude granting access under these provisions. Where co-development agreements or other legal restrictions prevent companies from sharing particular data, companies will work with qualified requestors to provide summary information where possible.
