# Correction: Spartalizumab in combination with platinum-doublet chemotherapy with or without canakinumab in patients with PD-L1-unselected, metastatic NSCLC

**DOI:** 10.1186/s12885-024-13210-9

**Published:** 2024-11-21

**Authors:** Armando Santoro, Garrido Pilar, Daniel S.W. Tan, Jon Zugazagoitia, Frances A. Shepherd, Alessandra Bearz, Fabrice Barlesi, Tae Min Kim, Tobias R. Overbeck, Enriqueta Felip, Can Cai, Simantini Eddy, Tracey McCulloch, Eric S. Schaefer

**Affiliations:** 1 Department of Biomedical Sciences, Humanitas University, Milan, Italy; 2 Department of Oncology and Hematology, IRCCS Humanitas Research Hospital, Humanitas Cancer Center, Via Manzoni 56, Rozzano, Milan 20089 Italy; 3 Department of Medical Oncology, Hospital Ramón Y Cajal, Madrid, Spain; 4 Department of Medical Oncology, National Cancer Center Singapore, Singapore, Singapore; 5 Department of Medical Oncology, University Hospital, 12 de Octubre, Madrid, Spain; 6 Department of Medical Oncology and Hematology, Princess Margaret Cancer Centre, Toronto, ON Canada; 7 Department of Medical Oncology, Centro Di Riferimento Oncologico - CRO, Aviano, Italy; 8 Department of Multidisciplinary Oncology and Therapeutic Innovations, Aix Marseille University, CNRS, INSERM, CRCM, APHM, CEPCM, CLIP, Marseille, France; 9 Faculté de Médecine, Université Paris Saclay, Kremlin Bicêtre, France; 10 Medical Oncology Department, Gustave Roussy, Villejuif, France; 11 Department of Internal Medicine, Seoul National University Hospital, Seoul, South Korea; 12 Department of Hematology and Medical Oncology, University Medical Center Göttingen, Göttingen, Germany; 13 Department of Medical Oncology Service, Vall d’Hebron University Hospital and Vall d’Hebron Institute of Oncology, Barcelona, Spain; 14 Novartis Pharmaceuticals Corporation, East Hanover, NJ USA; 15 Novartis Pharma AG, Basel, Switzerland; 16 Department of Medical Oncology, Highlands Oncology Group, Fayetteville, AZ USA

**Correction** **:** **BMC Cancer 24, 1307 (2024)**

**https://doi.org/10.1186/s12885-024-12841-2**

Following publication of the original article [1], the authors identified an error in the author name of Simantini Eddy. Their given name and family name were erroneously transposed.

The incorrect author name is: Eddy Simantini

The correct author name is: Simantini Eddy

The author group has been updated above and the original article [1] has been corrected.
