# Supplementary material for: Spartalizumab in combination with platinum-doublet chemotherapy with or without canakinumab in patients with PD-L1-unselected, metastatic NSCLC
Source: BMC Cancer. 2024 Oct 24;24:1307. doi: 10.1186/s12885-024-12841-2 (PMC11515544; doi:10.1186/s12885-024-12841-2)
Supplement: Supplementary file 1 — Supplementary Material 1 [file 12885_2024_12841_MOESM1_ESM.docx]

### Supplementary Data

**Spartalizumab in combination with platinum-doublet chemotherapy with or without canakinumab in patients with PD-L1-unselected, metastatic NSCLC**

Armando Santoro, Garrido Pilar, Daniel S.W. Tan, Luis Paz-Ares, Frances A. Shepherd, Alessandra Bearz, Fabrice Barlesi, Tae Min Kim, Tobias R. Overbeck, Enriqueta Felip, Cai Can, Eddy Simantini, Tracey McCulloch, Eric S Schaefer

### Methods

### Analysis sets

**Full-analysis set (FAS):** The FAS included all patients who received one dose of any component drug of the study treatment.

**Safety set:** The safety set comprised all patients who received at least one dose of any study drug of the treatment. Patients were analyzed according to the study treatment actually received, where actual treatment received was defined as:

- The assigned treatment if patients took at least one dose of that treatment
- The first treatment received if the assigned treatment was never received

**Dose determining set (DDS):** The DDS included all patients from the FAS who completed the minimum exposure criteria and had sufficient safety evaluations or experienced a dose-limiting toxicity (DLT) during the first 42 days of dosing.

The patients considered for the analysis of DLTs were:

- Patients experienced a DLT during the first 42 days of dosing
- Patients who did not have a DLT and had sufficient safety evaluations for ≥42 days following the commencement of study treatment

A patient was considered to have met the minimum exposure criteria if the patient received the first 2 doses of spartalizumab (SPARTA) and >50% of the planned dose of each chemotherapy for groups A, B and C, or the first two doses of SPARTA, first two doses of canakinumab (CAN) and >50% of the planned dose of each chemotherapy for group E.

**Pharmacokinetic analysis set (PAS):** The PAS included all patients who provided an evaluable pharmacokinetic (PK) profile. A profile was considered evaluable if all the following conditions were satisfied:

- A patient had received a planned dose of SPARTA prior to sampling
- For pre-dose samples, the sample was collected before the next dose administration
- For end-of-infusion samples, the sample was collected within 2 hours post end of infusion

The PAS of the platinum-doublet chemotherapy (PDC) agents (PAS for each agent: PAS-cisplatin, PAS-gemcitabine, PAS-pemetrexed, PAS-carboplatin, PAS-paclitaxel) included all patients who provided at least one evaluable PK concentration for the respective agent. A profile was considered evaluable if all the following conditions were satisfied:

- A patient had received a planned dose of PDC agent prior to sampling
- For pre-dose samples, the sample was collected before the next dose administration
- For samples scheduled to be taken prior to end of infusion (EOI), the samples were collected prior to EOI
- For post-EOI samples, the samples were collected post EOI

The CAN PAS included all patients who provided at least one evaluable CAN PK concentration. A profile was considered evaluable if all the following conditions were satisfied:

- Pre-dose samples were collected before the next dose administration
- Pre-dose samples (except for day 1 of cycle 1) were collected between 16 days (384 hours) and 26 days (624 hours) after the last 200 mg CAN dose administration
- A patient had received 200 mg of CAN prior to the post-dose PK sampling

**Immunogenicity (IG) analysis sets:**

SPARTA: The SPARTA IG prevalence set included all patients in the FAS with a determinant baseline IG sample or at least one determinant post-baseline IG sample.

The SPARTA IG incidence set included all patients in the IG prevalence set with a determinant baseline IG sample and at least one determinant post-baseline IG sample.

CAN: There was no IG analysis set of CAN and IG data was summarised using the safety set.

**Permitted dose adjustments and interruptions of study treatment**

For patients who did not tolerate the protocol-specified dosing schedule, dose interruptions and reductions were mandated to allow the patients to continue the study treatment.

Dose interruption for SPARTA and CAN included delaying or withholding the treatment for any reason (e.g., due to an adverse event [AE]) as well as interruption of treatment during an infusion.

If SPARTA, CAN, or PDC was interrupted because of an AE, the treatment for all study drugs was delayed up to a maximum of 2 weeks. After 2 weeks if the AE resolved, the only permitted study medication were allowed to resume.

Interruption of chemotherapy was not permitted for >6 weeks. Impeding chemotherapy was resumed after 6 weeks and for patients who did not recover from toxicities, chemotherapy was discontinued.\

**Ethics approval and consent to participate**

The trial has been approved by local ethic authorities for each centre as follows:

1. University Health Network Research Ethics Board, Toronto, Canada
2. Universität Ulm Ethik Kommission, Ulm, Germany
3. Universitätsklinikum Jena Ethik-Kommission, Jena, Germany
4. Ethics Committee of Georg-August-University of Göttingen, Göttingen, Germany
5. Ethik Kommission der Medizinischen Fakultät der Universität Duisburg-Essen, Essen, Germany
6. CEIm HM HOSPITALES, Madrid, Spain
7. HM Universitario Montepríncipe, Madrid, Spain
8. Comité de Protection des Personnes Ile-de France VII, Kremlin Bicêtre, France
9. The University of Texas MD Anderson Cancer Center Institutional Review Board, Houston, USA
10. Dana-Farber Cancer Institute IRB, Boston, USA
11. John Hopkins Medicine Office of Human Subjects Research-Institutional Review Board, Baltimore, USA
12. IntegReview Ethical Review Board, Austin, USA
13. University of Utah Institutional Review Board, Salt Lake City, USA
14. OHSU Institutional Review Board, Portland, USA
15. The Ethics Committee of the Leiden University Medical Center (CME LUMC) Effective as of 01 June 2019, Medisch Etische Toetsings Commissie Leiden – Den Haag – Delft (METC-LDD), Leiden, Netherlands
16. Research Ethics Committee National Taiwan University Hospital, Taipei, Taiwan
17. Institutional Review Board, National Cheng Kung University Hospital, Tainan, Taiwan
18. Baskent Universitesi Klinik Arastirmalar Etik Kurulu, Ankara, Turkey
19. Institutional Review Board, Faculty of Medicine, Chulalongkorn University, Patumwan Bangkok, Thailand
20. Office of Human Research Ethics Committee, Faculty of Medicine, Prince of Songkla University, HatYai Songkhla, Thailand
21. Institutional Review Board, Seoul National University Hospital, Seoul, South Korea
22. Ethics Committee- Hotel Dieu De France, Beirut, Lebanon
23. REK sør-øst komite B, Oslo, Norway
24. Medical Research Council Ethics Committee for Clinical Pharmacology, Budapest, Hungary
25. Komisja Bioetyczna przy Okręgowej Radzie Lekarskiej, Wielkopolskiej Izby Lekarskiej, Poznan, Poland
26. Comitato Etico Indipendente IRCCS Istituto Nazionale Tumori fondazione Pascale, Napoli, Italy
27. Comitato Etico IRCCS Ospedale San Raffaele di Milano, Milan, Italy
28. Comitato Etico Indipendente di area Vasta Emilia Centro, Bologna, Italy
29. Comitato Etica Indipendente IRCCS Istituto Clinico Humanitas, Rozzano, Italy
30. Comitato Etico dell’area Vasta Emilia Nord, Modena, Italy

**BLRM model for Groups A, B, and C**

For a triple combination of PDR001 with platinum doublets the 10-parameter BLRM comprises single agent dose-DLT relationships parts, which allow the incorporation of historical single-agent toxicity data, and an interaction part.

**Single agent parts**

Let π_1_(d_1_) be the risk of DLT for PDR001 given as a single agent Q3W at dose d_1_; π_2_(d_2_) be the risk of DLT for chemotherapy component 1 given as a single agent at a total daily dose of d_2_; π_3_(d_3_) be the risk of DLT for chemotherapy component 2 given as a single agent at a total daily dose of d_3_.

These single agent dose-DLT models are logistic:

PDR001 (Q3W): logit(π_1_(d_1_)) = log(α_1_) + β_1_ log(d_1_/ d_1_*)

Chemotherapy 1: logit(π_2_(d_2_)) = log(α_2_) + β_2_log(d_2_/d_2_*)

Chemotherapy 2: logit(π_3_(d_3_)) = log(α_3_) + β3log(d_3_/d_3_*)

where logit(π.(d.)) = log[π.(d.)/{1- π.(d.)}], d_1_* = 300mg is the reference doses of PDR001 and d_2_*, d_3_* are the reference dose for chemotherapy 1 and chemotherapy 2 of the platinum-doublets in each group respectively, α_1_, α_2_, α_3_, β_1_, β_2_, β_3_ > 0 and η_12_, η_13_, η_23_, η_123_ are the interaction coefficient.

**Interaction**

Under no interaction, the risk of a DLT for the kth model (k =1, 2, 3) at dose *d*_1_ of PDR001, dose *d*_2_ of chemotherapy 1, and dose *d*_3_ of chemotherapy 2 is:


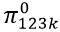
 (*d*_1_,*d*_2_,*d*_3_) = 1 - (1 - π_1_*_k_* (*d*_1_))(1 - π_2_*_k_* (*d*_2_))(1 - π_3_*_k_* (*d*_3_))

To allow for interaction between two chemotherapy components, and PDR001, odds multipliers are introduced:

| *η*_12_*_k_* | Two-way interaction between PDR001 and chemotherapy 1 |
| --- | --- |
| *η*_13_*_k_* | Two-way interaction between PDR001 and chemotherapy 2 |
| *η*_23_*_k_* | Two-way interaction between chemotherapy 1 and chemotherapy 2 |
| *η*_123_*_k_* | Three-way interaction between PDR001, chemotherapy 1, and chemotherapy 2 |

The risk of DLT for combination dose (*d*_1_,*d*_2_,*d*_3_) is then given by:

| odds (π_123_*_k_* (*d*_1_,*d*_2_,*d*_3_)) | = | exp (*η*_12_*_k_* × *d*_1_ / *d*_1_ * × *d*_2_ / *d*_2_ *) | |
| --- | --- | --- | --- |
|  |  | × | exp (*η*_13_*_k_* × *d*_1_ / *d*_1_ * × *d*_3_ / *d*_3_ *) |
|  |  | × | exp (*η*_23_*_k_* × *d*_2_ / *d*_2_ * × *d*_3_ / *d*_3_ *) |
|  |  | × | exp (*η*_123_*_k_* × *d*_1_ / *d*_1_ * × *d*_2_ / *d*_2_ * × *d*_3_ / *d*_3_ *) |
|  |  | × | odds (π^0^_123k_ (*d*_1_,*d*_2_,*d*_3_)) |

where odds(π) = π/ (1 - π); *ƞ_ijk_* is the kth model log-odds ratio between the interaction and no interaction model at the reference dose for treatments i and j and a zero dose of the third treatment; and (*ƞ*_12_*_k_* + *ƞ*_23_*_k_* + *ƞ*_32_*_k_* + *ƞ*_123_*_k_*) is the kth model log-odds ratio between the interaction and no interaction model at the reference dose for all three treatments. Here *ƞ_ijk_* = 0 corresponds to no interaction, with *ƞ_ijk_* > 0 and *ƞ_ijk_* < 0 representing synergistic and antagonistic toxicity respectively.

**Prior specifications**

The Bayesian approach requires the specification of prior distributions for all model parameters which include, for the kth model (k = 1, 2, 3), the single agent parameters log(α_1k_) and log(β_1k_) for PDR001, log(α_2k_) and log(β_2k_) for chemotherapy 1, log(α_3k_) andlog(β_3k_) for chemotherapy 2, and interaction parameters *ƞ*_12_*_k_*, *ƞ*_13_*_k_*, *ƞ*_23_*_k_*, and *ƞ*_123_*_k_*. A meta-analytic-predictive (MAP) approach is used to derive a prior distribution for the single-agent model parameters based upon available DLT data. For chemotherapy 1 and chemotherapy 2, 100% weakly informative prior will be used. For PDR001, this is then robustified by creating a mixture prior including both a component derived from the MAP prior and a weakly informative robustification component. This robustification allows for the possibility that the dose/toxicity relationship for PDR001 in combination differs substantially from that of the single agent.

**Prior distribution for the logistic parameters**

**Description of the meta-analytic-predictive (MAP) approach**

The aim of the MAP approach is to derive a prior distribution for the logistic parameters (log(*α*^*^), log(*β*^*^)) of the new trial using DLT data from historical studies.

Let r_ds_ and n_ds_ be the number of patients with a DLT, and the total number of patients at dose d in historical trial s (s = 1,…,S). The corresponding probability of a DLT is π_ds_. The model specifications for the derivation of the MAP prior are as follows:

r_ds_ | π_ds_ ~Bin(π_ds_, n_ds_)

logit(π_ds_) = log(α_s_) + β_s_ log (d/d_ref_)

(log(α_s_), log(β_s_)) | µ, ψ ~ BVN(µ, ψ), s = 1,…, S

(log(α^*^), log(β^*^)) | µ, ψ ~ BVN(µ, ψ)

Where d_ref_ is the reference dose. The parameters µ=(µ_1_, µ_2_) and ψ are the mean and between-trial covariance matrix for the logistic parameters, the latter with standard deviations τ_1_, τ_2_, and correlation ρ. The parameters τ_1_ and τ_2_ quantify the degree of between trial heterogeneity. The following priors are used for these parameters:

- normal priors for µ_1_ and µ_2_,
- log-normal priors for τ_1_ and τ_2_, and
- a uniform prior for ρ.

The MAP prior for single-agent model parameters in the new trial, (log(α^*^), log(β^*^)), is the predictive distribution

(log(α^*^), log(β^*^)) | (r_ds_, n_ds_: s = 1,…,S)

Since the predictive distribution is not available analytically, MCMC is used to simulate values from this distribution. This is implemented using JAGS version 3.4.0. The sample from this distribution is then approximated by a mixture of bivariate normal (BVN) distributions. BVN mixtures with increasing numbers of mixture components are fitted to the sample using the expectation-maximization (EM) algorithm *(1)*. The optimal number of components of the mixture is then identified using the Akaike information criterion (AIC) *(2)*.

**Single agent PDR001 Q3W**

The prior distribution of PDR001 Q4W single agent BLRM model parameters (log(α_1_), log(β_1_)) is a mixture of two components: a MAP component and a robustification component used to allow for a dose/toxicity relationship for PDR001 in combination that differs substantially from that in single agent. The components form a mixture prior with respective weights (0.95, 0.05).

**MAP Component:**

For the MAP model for PDR001, reference dose d_ref_ = 300 mg (Q3W) is used, and data from S = 1 historical study is available.

Weakly informative normal priors are assumed for µ_1_ and µ_2_, with means corresponding to an assumed 10% risk of DLT at the reference dose of 300 mg, and a doubling increment in dose leading to a doubling in the odds of a DLT, respectively. Priors for τ_1_ and τ_2_ are assigned such that (1) their medians correspond to moderate between trial heterogeneity, and (2) their uncertainty (95% prior interval) cover plausible between-trial standard deviations. *(3)*

The prior distributions for the model used for deriving the MAP priors are specified in the table below

**Prior distributions for the parameters of the MAP model used to derive the prior for the single-agent PDR001 model parameters**

| **Parameter** | **Prior distribution** |
| --- | --- |
| µ_1_ | N(mean = logit(0.1), sd = 2) |
| µ_2_ | N(mean = logit(0.5), sd=1) |
| τ_1_ | log-normal(mean = 0.250, sd = log(2)/1.96) |
| τ_2_ | log-normal(mean = 0.125, sd = log(2)/1.96) |
| ρ | uniform(-1,1) |

**Historical data**

The dose-DLT data of PDR001 single agent from the following clinical study are considered as the relevant information and used to derive the prior distribution for the BLRM parameters (log(α_1_), log(β_1_)).

- **CPDR001X2101:** open label multicenter Phase I/II study of the safety and efficacy of PDR001 administered to patients with advanced malignancies.

**Robustification component:**

To take into account the potential situation that PDR001 in combination is substantially more toxic than when administered as single agent, and that the longer DLT period of 42 days may increase the chance of experiencing toxicity, a second prior component with vague bivariate normal distribution centered around higher toxicity is added to improve the robustness of the final prior. The parameters of this weakly informative prior distribution are described below:

1. The mean (log(α_1_), log(β_1_)) = (logit(0.33), 0), i.e. the median DLT rate at the reference dose (300 mg) was assumed to be 0.33 and doubling in dose was assumed to double odds of DLT.
2. To complete the specification, the prior standard deviation, sd(log(α_1_), log(β_1_)), was set equal to (2,1), which allows for considerably prior uncertainty for the dose-toxicity profile.
3. The correlation, corr(log(α_1_), log(β_1_)), was set equal to 0.

**Single agent chemotherapy 1 or chemotherapy 2**

We use weakly-informative priors since we have no relevant evidence (data) at hand for the DLT of single agent chemotherapies for this population. The parameters of this weakly informative prior distribution are described below:

- Weakly informative normal priors are assumed for µ_1_ and µ_2_, with means corresponding to an assumed 1% risk of DLT at the reference doses within 42 days of DLT period for chemotherapy 1 and chemotherapy 2 respectively, and a 2-fold increment in dose leading to a doubling in the odds of a DLT, respectively.
- To complete the specification, the prior standard deviation, sd(log(α_1_), log(β_1_)), was set equal to (2,1), which allows for considerably prior uncertainty for the dose-toxicity profile.
- The correlation, corr(log(α_1_), log(β_1_)), was set equal to 0.

**Prior distribution for the interaction parameter**

Based on available information, there is an antagonistic or synergistic interaction between chemotherapy 1 and chemotherapy 2 of the platinum doublets for Group A, B and C. No interaction is expected for the other two-way combinations, and for the three-way combination no interaction is expected over and above that already described. However, in all cases considerable uncertainty remains as to the true interaction, and normal priors are used that allow for both synergistic and antagonistic toxicity. The following assumptions will be used for interaction parameters for model k (k = 1, 2, 3).

**Chemotherapy 1 and chemotherapy 2 for each group of A, B and C:**

- *ƞ*_23_*_k_* is normally distributed and centered at 1.1, i.e. 10% increase in odds of DLT over independence at the combination starting dose
- 97.5^th^ percentile is log(3), i.e. 3-fold increase in odds of DLT over independence at the combination starting dose

**Chemotherapy 1 (or 2) and PDR001:**

- *ƞ*_12_*_k_*, *ƞ*_13_*_k_* are normally distributed and centered at 0, i.e. 0% increase in odds of DLT over independence at the combination starting dose
- 97.5^th^ percentile is log(2), i.e. 2-fold increase in odds of DLT over independence at the combination starting dose

**Chemotherapy 1, chemotherapy 2 and PDR001:**

- *ƞ*_123_*_k_* is normally distributed and centered at 0, i.e. 0% increase in odds of DLT over independence at the combination starting dose
- 97.5^th^ percentile is log(1.5), i.e. 1.5-fold increase in odds of DLT over independence at the combination starting dose

**BLRM model for Group E**

**Statistical model**

For a “triple” combination of PDR001, canakinumab, and platinum-doublet chemotherapy (pemetrexed/cisplatin) in group E, the 10-parameter Bayesian logistic regression model (BLRM) is implemented to determine the relationship between the DLTs and dose levels. The model comprises single agent dose-DLT relationships parts, which allow the incorporation of historical single-agent toxicity data, and an interaction part.

**Single agent parts**

Let π_1_(d_1_) be the risk of DLT for PDR001 given as a single agent Q3W at dose d_1_; π_2_(d_2_) be the risk of DLT for canakinumab given as a single agent Q3W at dose of d_2_; π_3_(d_3_) be the risk of DLT for platinum-doublet chemotherapy at a total daily dose of d_3_.

These single agent dose-DLT models are logistic:

PDR001 (Q3W): logit(π_1_(d_1_)) = log(α_1_) + β_1_ log(d_1_/d_1_*)

Canakinumab (Q3W): logit(π_2_(d_2_)) = log(α_2_) + β_2_ log(d_2_/d_2_*)

platinum-doublet chemotherapy: logit(π_3_(d_3_)) = log(α_3_) + β_3_ log(d_3_/d_3_*)

Where logit (π.(d.)) = log[π.(d.)/{1- π.(d.)}], d_1_* is the reference doses of PDR001 and d_2_*, d_3_* are the reference dose for canakinumab and platinum-doublet chemotherapy (pemetrexed/cisplatin) of the platinum-doublets in each group respectively, α_1_, α_2_, α_3_, β_1_, β_2_, β_3_ > 0 and η_12_, η_13_, η_23_, η_123_ are the interaction coefficients.

**Interaction**

Under no interaction, the risk of a DLT for the model at dose *d*_1_ of PDR001, dose *d*_2_ of canakinumab, and dose *d*_3_ of platinum-doublet chemotherapy (pemetrexed/cisplatin) is:

π^0^_123_ (*d*_1_,*d*_2_,*d*_3_) = 1 - (1 - π_1_ (*d*_1_))(1 - π_2_ (*d*_2_))(1 - π_3_ (*d*_3_))

To allow for interaction between PDR001, canakinumab and platinum-doublet chemotherapy, odds multipliers are introduced:

| *η*_12_ | Two-way interaction between PDR001 and Canakinumab |
| --- | --- |
| *η*_13_ | Two-way interaction between PDR001 and platinum-doublet chemotherapy |
| *η*_23_ | Two-way interaction between Canakinumab and platinum-doublet chemotherapy |
| *η*_123_ | Three-way interaction between PDR001, Canakinumab and platinum-doublet chemotherapy |

The risk of DLT for combination dose (*d*_1_,*d*_2_,*d*_3_) is then given by:

| odds (π_123_ (*d*_1_,*d*_2_,*d*_3_)) | = | exp (*η*_12_ × *d*_1_ / *d*_1_ * × *d*_2_ / *d*_2_ *) | |
| --- | --- | --- | --- |
|  |  | × | exp (*η*_13_ × *d*_1_ / *d*_1_ * × *d*_3_ / *d*_3_ *) |
|  |  | × | exp (*η*_23_ × *d*_2_ / *d*_2_ * × *d*_3_ / *d*_3_ *) |
|  |  | × | exp (*η*_123_ × *d*_1_ / *d*_1_ * × *d*_2_ / *d*_2_ * × *d*_3_ / *d*_3_ *) |
|  |  | × | odds (π^0^_123_ (*d*_1_,*d*_2_,*d*_3_)) |

where odds(π) = π/ (1 - π); *ƞ_ij_* is the model log-odds ratio between the interaction and no interaction model at the reference dose for treatments i and j and a zero dose of the third treatment; and (*ƞ*_12_ + *ƞ*_23_ + *ƞ*_32_ + *ƞ*_123_) is the log-odds ratio between the interaction and no interaction model at the reference dose for all three treatments. Here *ƞ_ij_* = 0 corresponds to no interaction, with *ƞ_ij_* > 0 and *ƞ_ij_* < 0 representing synergistic and antagonistic toxicity respectively.

**Weakly prior specifications**

The Bayesian approach requires the specification of prior distributions for all model parameters which include the single agent parameters log(α_1_) and log(β_1_) for PDR001, log(α_2_) and log(β_2_) for canakinumab, log(α_3_) and log(β_3_) for platinum-doublet chemotherapy, and interaction parameters *ƞ*_12_, *ƞ*_13_, *ƞ*_23_, and *ƞ*_123_. A meta-analytic-approach is used to derive a prior distribution for all model parameters based upon available DLT data.

Weakly informative prior distribution for single agents

The same weakly-informative as the initial prior is used for PDR001, canakinumab, and platinum-doublet chemotherapy. The parameters of this weakly informative prior distribution are described below:

- Weakly informative normal priors are assumed for µ_1_ and µ_2_, with means corresponding to an assumed 10 % risk of DLT at the reference doses within 42 days of DLT period for each agent, and a 2-fold increment in dose leading to a doubling in the odds of a DLT, respectively.
- To complete the specification, the prior standard deviation, sd(log(α_1_), log(β_1_)), was set equal to (2,1), which allows for considerably prior uncertainty for the dose-toxicity profile.
- The correlation, corr(log(α_1_), log(β_1_)), was set equal to 0.

Weakly prior distribution for the interaction parameters

Based on available information, there is no antagonistic or synergistic interaction between any two-way combinations, and the three-way combination of component drugs. However, in all cases considerable uncertainty remains as to the true interaction, and normal priors are used that allow for both synergistic and antagonistic toxicity. The following assumptions will be used for interaction parameters for the model.

**1) PDR001 and canakinumab:**

- *ƞ*_12_ is normally distributed and centered at 0, i.e. 0% increase in odds of DLT over independence at the combination starting dose
- 97.5^th^ percentile is log(2), i.e. 2-fold increase in odds of DLT over independence at the combination starting dose

**2) PDR001 and platinum-doublet chemotherapy:**

- *ƞ*_13_ is normally distributed and centered at 0, i.e. 0% increase in odds of DLT over independence at the combination starting dose
- 97.5^th^ percentile is log(2), i.e. 2-fold increase in odds of DLT over independence at the combination starting dose

**3) Platinum-doublet chemotherapy and canakinumab**

- *ƞ*_23_ is normally distributed and centered at 0, i.e. 0% increase in odds of DLT over independence at the combination starting dose
- 97.5^th^ percentile is log(2), i.e. 2-fold increase in odds of DLT over independence at the combination starting dose

**4) PDR001, canakinumab, and platinum-doublet chemotherapy:**

- *ƞ*_123_ is normally distributed and centered at 0, i.e. 0% increase in odds of DLT over independence at the combination starting dose
- 97.5^th^ percentile is log(1.5), i.e. 1.5-fold increase in odds of DLT over independence at the combination starting dose

**Historical data**

The dose-DLT data for single agent or the combination of agents from the following clinical studies are considered and will be incorporated into the prior model using Meta-analytic approaches.

- **CPDR001X2101:** An open label multicenter Phase I/II study of the safety and efficacy of PDR001 administered to patients with advanced malignancies.
- **CPDR001C2101 (group B)**, Phase Ib, multicenter, open label study of PDR001 in combination with platinum-doublet chemotherapy in PD-L1 unselected, metastatic NSCLC patients.
- **CPDR001X2103**, Phase Ib, open-label, multi-center study to characterize the safety, tolerability and pharmacodynamics of PDR001 in combination with CJM112, EGF816, Ilaris® (canakinumab) or Mekinist® (trametinib).

Prior distributions for the logistic parameters

The MAP prior for individual component drugs is derived from the composite prior by adjusting between-trial heterogeneity. The standard deviations for single agents are discounted moderately:


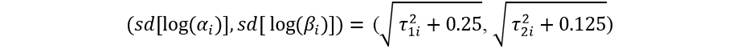


*i* =1, 2, 3 are for PDR001, canakinumab, platinum-doublet chemotherapy, respectively.

**References**

1. Rubin APDNMLDB. Maximum Likelihood from Incomplete Data via the EM Algorithm. Journal of the Royal Statistical Society Series B (Methodological). 1977;39:1-38.

2. Akaike H. A new look at the statistical model identification. IEEE Transactions on Automatic Control. 1974;19:716-723.

3. Neuenschwander B, Capkun-Niggli G, Branson M, Spiegelhalter DJ. Summarizing historical information on controls in clinical trials. Clinical Trials. 2010;7:5-18.

**Supplementary Figure S1:** Study design


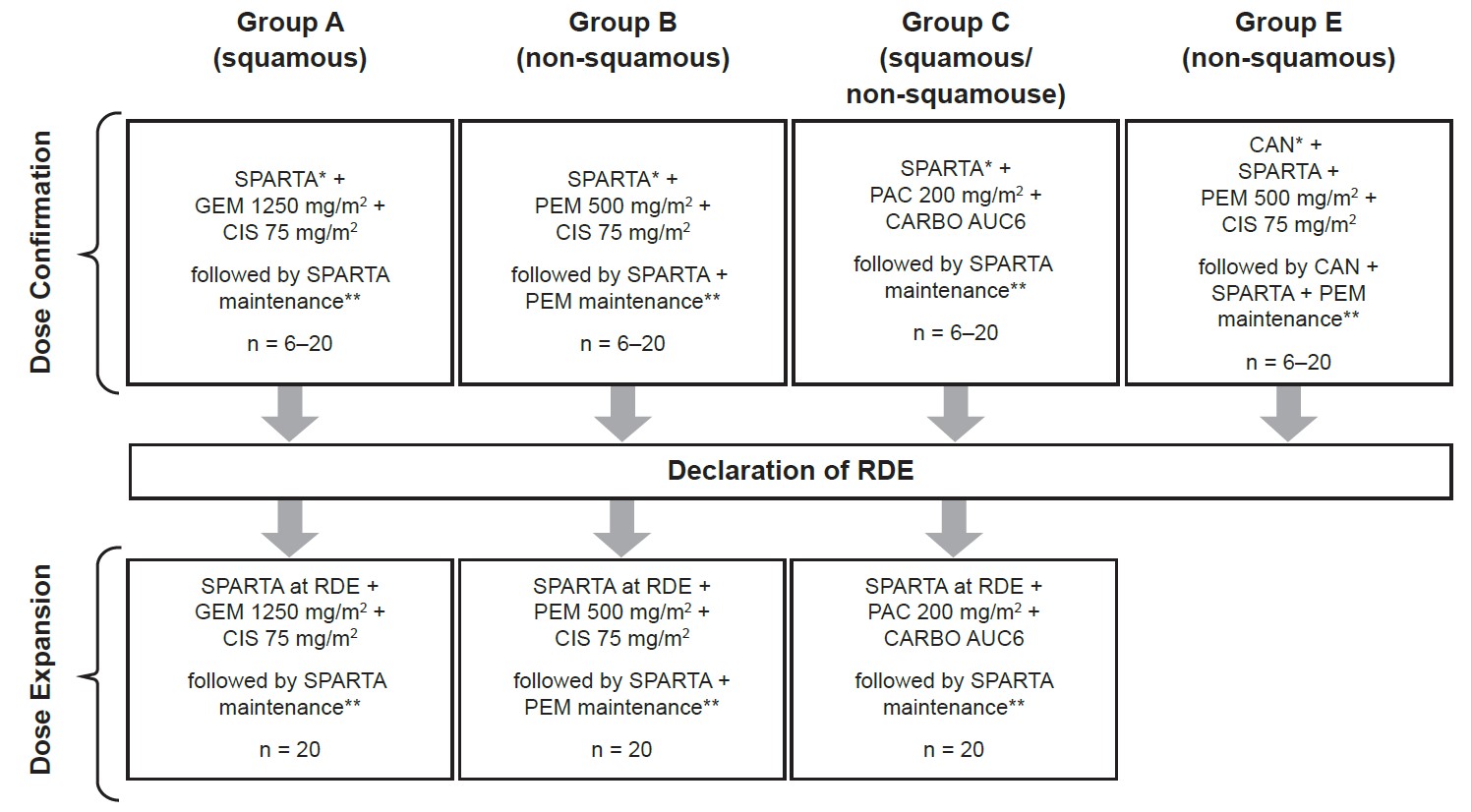


AUC, The area under the concentration-time curve; CAN, canakinumab; CARBO, carboplatin; CI, confidence interval; CIS, cisplatin; GEM, gemcitabine; PAC, paclitaxel; PEM, pemetrexed; RDE, recommended dose for expansion; SPARTA, spartalizumab.

*Dose Confirmation:

SPARTA Initial Dose Level: 300mg i.v. every 3 weeks

SPARTA Dose Level -1: 300mg i.v. every 6 weeks

CAN Initial Dose Level: 200mg s.c. every 3 weeks

CAN Dose Level -1: 200mg s.c. every 6 weeks

**Dose Expansion:

The starting dose of SPARTA +/- PEM during maintenance should be consistent with the last dose taken by the patient during Induction

**Supplementary Figure S2:** Patient Disposition


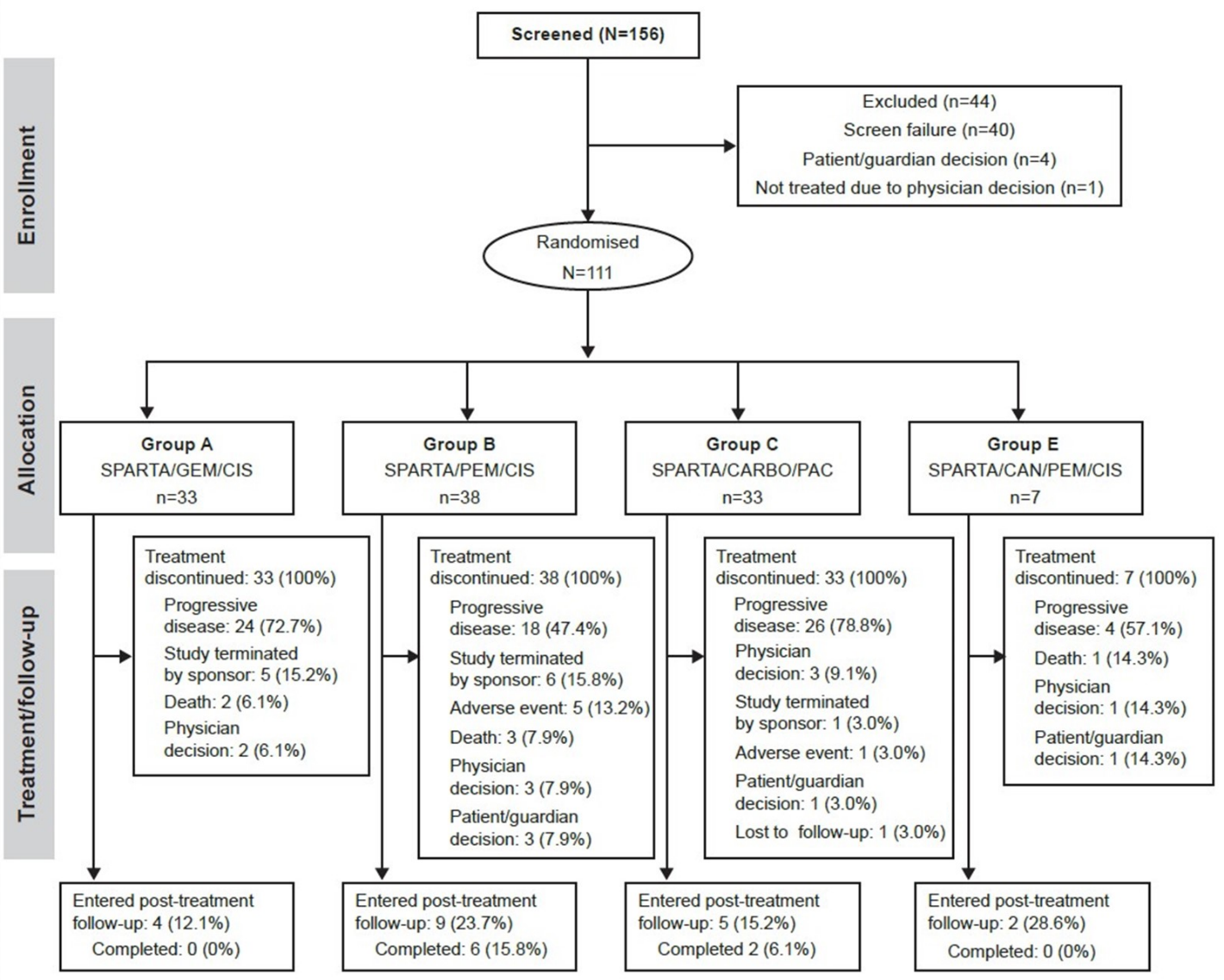


CAN, canakinumab; CARBO, carboplatin; CIS, cisplatin; GEM, gemcitabine; PAC, paclitaxel; PEM, pemetrexed; SPARTA, spartalizumab.

**Supplementary Table S1:** AEs (≥20% of patients) suspected to be study drug related by preferred term and group (safety analysis set)

| Preferred term | **Group A**  **(SPARTA/GEM/CIS) N=33** | | **Group B**  **(SPARTA/PEM/CIS) N=38** | | **Group C**  **(SPARTA/CARBO/PAC) N=33** | | **Group E**  **(SPARTA/CAN/PEM/CIS) N=7** | |
| --- | --- | --- | --- | --- | --- | --- | --- | --- |
|  | **All grades**  **n (%)** | **Grade 3/4**  **n (%)** | **All grades**  **n (%)** | **Grade 3/4**  **n (%)** | **All grades**  **n (%)** | **Grade 3/4**  **n (%)** | **All grades**  **n (%)** | **Grade 3/4**  **n (%)** |
| Patients with ≥1 AE | 32 (97.0) | 20 (60.6) | 38 (100) | 23 (60.5) | 32 (97.0) | 21 (63.6) | 7 (100) | 3 (42.9) |
| Neutropenia | 16 (48.5) | 9 (27.3) | 19 (50.0) | 13 (34.2) | 18 (54.5) | 15 (45.5) | 2 (28.6) | 1 (14.3) |
| Anemia | 14 (42.4) | 3 (9.1) | 15 (39.5) | 4 (10.5) | 14 (42.4) | 2 (6.1) | 3 (42.9) | 2 (28.6) |
| Nausea | 12 (36.4) | 0 | 27 (71.1) | 2 (5.3) | 12 (36.4) | 0 | 2 (28.6) | 0 |
| Asthenia | 10 (30.3) | 0 | 11 (28.9) | 0 | 13 (39.4) | 2 (6.1) | 0 | 0 |
| Fatigue | 8 (24.2) | 2 (6.1) | 7 (18.4) | 0 | 7 (21.2) | 0 | 4 (57.1) | 0 |
| Thrombocytopenia | 8 (24.2) | 5 (15.2) | 4 (10.5) | 2 (5.3) | 8 (24.2) | 4 (12.1) | 0 | 0 |
| Constipation | 6 (18.2) | 0 | 10 (26.3) | 0 | 8 (24.2) | 0 | 1 (14.3) | 0 |
| Diarrhea | 6 (18.2) | 0 | 7 (18.4) | 3 (7.9) | 9 (27.3) | 0 | 1 (14.3) | 0 |
| Alanine aminotransferase increased | 5 (15.2) | 0 | 5 (13.2) | 2 (5.3) | 7 (21.2) | 2 (6.1) | 0 | 0 |
| Alopecia | 4 (12.1) | 0 | 6 (15.8) | 0 | 9 (27.3) | 0 | 0 | 0 |
| Decreased appetite | 4 (12.1) | 0 | 6 (15.8) | 0 | 10 (30.3) | 0 | 4 (57.1) | 0 |
| Hypomagnesaemia | 4 (12.1) | 0 | 2 (5.3) | 0 | 7 (21.2) | 0 | 0 | 0 |
| Neuropathy peripheral | 4 (12.1) | 0 | 2 (5.3) | 0 | 7 (21.2) | 0 | 0 | 0 |
| Pruritus | 4 (12.1) | 0 | 9 (23.7) | 0 | 8 (24.2) | 0 | 0 | 0 |
| Rash | 4 (12.1) | 0 | 10 (26.3) | 0 | 7 (21.2) | 0 | 1 (14.3) | 0 |
| Arthralgia | 3 (9.1) | 0 | 2 (5.3) | 0 | 8 (24.2) | 0 | 0 | 0 |
| Vomiting | 3 (9.1) | 0 | 12 (31.6) | 3 (7.9) | 7 (21.2) | 0 | 1 (14.3) | 0 |
| Blood creatinine increased | 2 (6.1) | 0 | 10 (26.3) | 0 | 2 (6.1) | 0 | 3 (42.9) | 0 |
| Gamma-glutamyl transferase increased | 2 (6.1) | 0 | 4 (10.5) | 2 (5.3) | 7 (21.2) | 3 (9.1) | 0 | 0 |
| Oedema peripheral | 1 (3.0) | 0 | 8 (21.1) | 1 (2.6) | 1 (3.0) | 0 | 0 | 0 |
| Lacrimation increased | 0 | 0 | 8 (21.1) | 0 | 0 | 0 | 1 (14.3) | 0 |
| Myalgia | 0 | 0 | 2 (5.3) | 0 | 9 (27.3) | 0 | 1 (14.3) | 0 |

AE, adverse event; CAN, canakinumab; CARBO, carboplatin; CIS, cisplatin; GEM, gemcitabine; PAC, paclitaxel; PEM, pemetrexed; SPARTA, spartalizumab.

**Supplementary Table S2:** SAEs by preferred term (≥2% of patients) (safety analysis set)

| **Preferred term** | **Group A**  **(SPARTA/GEM/CIS) N=33**  **n (%)** | **Group B**  **(SPARTA/PEM/CIS) N=38**  **n (%)** | **Group C**  **(SPARTA/CARBO/PAC) N=33**  **n (%)** | **Group E**  **(SPARTA/CAN/PEM/CIS) N=7**  **n (%)** |
| --- | --- | --- | --- | --- |
| Patients with ≥1 SAE | 15 (45.5) | 22 (57.9) | 13 (39.4) | 4 (57.1) |
| Abdominal pain | 1 (3.0) | 0 | 0 | 0 |
| Acute kidney injury | 1 (3.0) | 2 (5.3) | 0 | 0 |
| Acute respiratory failure | 0 | 1 (2.6) | 1 (3.0) | 0 |
| Aphasia | 1 (3.0) | 0 | 0 | 0 |
| Bone pain | 0 | 0 | 1 (3.0) | 0 |
| Cardiac arrest | 0 | 2 (5.3) | 0 | 0 |
| Cerebral haemorrhage | 0 | 0 | 0 | 1 (14.3) |
| Cerebrovascular accident | 0 | 1 (2.6) | 1 (3.0) | 0 |
| Cerebrovascular disorder | 1 (3.0) | 0 | 0 | 0 |
| Cholecystitis | 0 | 0 | 0 | 1 (14.3) |
| Diarrhea | 0 | 2 (5.3) | 0 | 0 |
| Dyspnoea | 0 | 0 | 3 (9.1) | 0 |
| Enterocolitis | 0 | 0 | 0 | 1 (14.3) |
| Fall | 0 | 0 | 1 (3.0) | 0 |
| Febrile neutropenia | 1 (3.0) | 1 (2.6) | 2 (6.1) | 0 |
| Intestinal perforation | 0 | 0 | 1 (3.0) | 0 |
| Lipase increased | 1 (3.0) | 0 | 0 | 0 |
| Pancytopenia | 0 | 3 (7.9) | 0 | 0 |
| Pneumonia | 2 (6.1) | 1 (2.6) | 2 (6.1) | 1 (14.3) |
| Pulmonary embolism | 0 | 1 (2.6) | 1 (3.0) | 1 (14.3) |
| Pyrexia | 3 (9.1) | 0 | 0 | 0 |
| Respiratory tract infection | 2 (6.1) | 0 | 0 | 0 |
| Seizure | 1 (3.0) | 0 | 2 (6.1) | 0 |
| Vomiting | 0 | 3 (7.9) | 0 | 0 |

CAN, canakinumab; CARBO, carboplatin; CIS, cisplatin; GEM, gemcitabine; PAC, paclitaxel; PEM, pemetrexed; SAE, serious adverse event; SPARTA, spartalizumab.

**Supplementary Table S3:** SAEs suspected to be related to study treatment by preferred term (≥2% of patients) (safety analysis set)

| **Preferred term** | **Group A**  **(SPARTA/GEM/CIS) N=33**  **n (%)** | **Group B**  **(SPARTA/PEM/CIS) N=38**  **n (%)** | **Group C**  **(SPARTA/CARBO/PAC) N=33**  **n (%)** | **Group E**  **(SPARTA/CAN/PEM/CIS) N=7**  **n (%)** |
| --- | --- | --- | --- | --- |
| Patients with ≥1 SAE | 4 (12.1) | 14 (36.8) | 4 (12.1) | 0 (0.0) |
| Anemia | 0 | 1 (2.6) | 0 | 0 |
| Febrile neutropenia | 1 (3.0) | 1 (2.6) | 1 (3.0) | 0 |
| Leukopenia | 0 | 1 (2.6) | 0 | 0 |
| Pancytopenia | 0 | 3 (7.9) | 0 | 0 |
| Cardiotoxicity | 0 | 1 (2.6) | 0 | 0 |
| Abdominal pain | 1 (3.0) | 0 | 0 | 0 |
| Diarrhea | 0 | 2 (5.3) | 0 | 0 |
| Nausea | 0 | 1 (2.6) | 0 | 0 |
| Neutropenic colitis | 0 | 0 | 1 (3.0) | 0 |
| Vomiting | 0 | 2 (5.3) | 0 | 0 |
| Mucosal inflammation | 0 | 0 | 1 (3.0) | 0 |
| Pyrexia | 1 (3.0) | 0 | 0 | 0 |
| Pneumonia | 0 | 1 (2.6) | 0 | 0 |
| Sepsis | 0 | 1 (2.6) | 0 | 0 |
| Urinary tract infection | 0 | 1 (2.6) | 0 | 0 |
| Lipase increased | 1 (3.0) | 0 | 0 | 0 |
| Hyponatremia | 0 | 1 (2.6) | 0 | 0 |
| Cerebrovascular accident | 0 | 1 (2.6) | 0 | 0 |
| Posterior reversible encephalopathy syndrome | 0 | 1 (2.6) | 0 | 0 |
| Acute kidney injury | 0 | 2 (5.3) | 0 | 0 |
| Nephropathy | 0 | 1 (2.6) | 0 | 0 |
| Dyspnea | 0 | 0 | 1 (3.0) | 0 |

CAN, canakinumab; CARBO, carboplatin; CIS, cisplatin; GEM, gemcitabine; PAC, paclitaxel; PEM, pemetrexed; SAE, serious adverse event; SPARTA, spartalizumab.

**Supplementary Table S4:** AEs leading to dose adjustment or dose interruption (≥10% of patients), by preferred term (Safety analysis set)

|  | **Group A** | | **Group B** | | **Group C** | | **Group E** | |
| --- | --- | --- | --- | --- | --- | --- | --- | --- |
|  | **(SPARTA/GEM/CIS)** | | **(SPARTA/PEM/CIS)** | | **(SPARTA/CARBO/PAC)** | | **(SPARTA/CAN/PEM/CIS)** | |
|  | **N=33** | | **N=38** | | **N=33** | | **N=7** | |
|  | **Any grade** | **Grade 3/4** | **Any grade** | **Grade 3/4** | **Any grade** | **Grade 3/4** | **Any grade** | **Grade 3/4** |
| **Preferred Term** | **n (%)** | **n (%)** | **n (%)** | **n (%)** | **n (%)** | **n (%)** | **n (%)** | **n (%)** |
| Patients with ≥1 AE | 26 (78.8) | 14 (42.4) | 22 (57.9) | 11 (28.9) | 20 (60.6) | 9 (27.3) | 5 (71.4) | 2 (28.6) |
| Neutropenia | 9 (27.3) | 5 (15.2) | 7 (18.4) | 2 (5.3) | 3 (9.1) | 1 (3.0) | 1 (14.3) | 1 (14.3) |
| Pneumonitis | 4 (12.1) | 0 | 2 (5.3) | 0 | 3 (9.1) | 0 | 0 | 0 |
| Pyrexia | 4 (12.1) | 0 | 0 | 0 | 0 | 0 | 0 | 0 |
| Fatigue | 3 (9.1) | 1 (3.0) | 0 | 0 | 0 | 0 | 1 (14.3) | 0 |
| Anaemia | 2 (6.1) | 1 (3.0) | 5 (13.2) | 1 (2.6) | 1 (3.0) | 0 | 0 | 0 |
| Blood creatinine increased | 1 (3.0) | 0 | 6 (15.8) | 0 | 0 | 0 | 2 (28.6) | 0 |
| Peripheral oedema | 0 | 0 | 4 (10.5) | 1 (2.6) | 0 | 0 | 0 | 0 |
| Nausea | 1 (3.0) | 0 | 2 (5.3) | 0 | 0 | 0 | 1 (14.3) | 0 |
| Cholecystitis | 0 | 0 | 0 | 0 | 0 | 0 | 1 (14.3) | 1 (14.3) |
| Diarrhoea | 0 | 0 | 1 (2.6) | 0 | 0 | 0 | 1 (14.3) | 0 |
| COPD | 0 | 0 | 0 | 0 | 0 | 0 | 1 (14.3) | 0 |
| Stomatitis | 0 | 0 | 0 | 0 | 0 | 0 | 1 (14.3) | 0 |

AE, adverse event; CAN, canakinumab; CARBO, carboplatin; CIS, cisplatin; COPD Chronic obstructive pulmonary disease; GEM, gemcitabine; PAC, paclitaxel; PEM, pemetrexed; SPARTA, spartalizumab.

**Supplementary Table S5:** AEs leading to dose discontinuation (≥2% of patients), by preferred term (Safety analysis set)

|  | **Group A**  **(SPARTA/GEM/CIS)**  **N=33** | | **Group B (SPARTA/PEM/CIS)**  **N=38** | | **Group C (SPARTA/CARBO/PAC) N=33** | | **Group E (SPARTA/CAN/PEM/CIS) N=7** | |
| --- | --- | --- | --- | --- | --- | --- | --- | --- |
|  | **All grades** | **Grade 3/4** | **All grades** | **Grade 3/4** | **All grades** | **Grade 3/4** | **All grades** | **Grade 3/4** |
| **Preferred Term** | **n (%)** | **n (%)** | **n (%)** | **n (%)** | **n (%)** | **n (%)** | **n (%)** | **n (%)** |
| Patients with ≥1 AE | 4 (12.1) | 1 (3.0) | 13 (34.2) | 4 (10.5) | 3 (9.1) | 1 (3.0) | 3 (42.9) | 0 |
| ALT increased | 1 (3.0) | 1 (3.0) | 0 | 0 | 0 | 0 | 0 | 0 |
| AST increased | 1 (3.0) | 0 | 0 | 0 | 0 | 0 | 0 | 0 |
| Blood creatinine increased | 1 (3.0) | 0 | 5 (13.2) | 0 | 0 | 0 | 0 | 0 |
| Implant site dehiscence | 1 (3.0) | 0 | 0 | 0 | 0 | 0 | 0 | 0 |
| Septic shock | 1 (3.0) | 0 | 0 | 0 | 0 | 0 | 0 | 0 |
| Tinnitus | 1 (3.0) | 0 | 0 | 0 | 0 | 0 | 0 | 0 |
| Acute kidney injury | 0 | 0 | 2 (5.3) | 1 (2.6) | 0 | 0 | 0 | 0 |
| Anemia | 0 | 0 | 1 (2.6) | 1 (2.6) | 0 | 0 | 0 | 0 |
| Asthenia | 0 | 0 | 1 (2.6) | 1 (2.6) | 0 | 0 | 0 | 0 |
| Cardiac arrest | 0 | 0 | 1 (2.6) | 0 | 0 | 0 | 0 | 0 |
| Cardiotoxicity | 0 | 0 | 1 (2.6) | 0 | 0 | 0 | 0 | 0 |
| Intestinal perforation | 0 | 0 | 0 | 0 | 1 (3.0) | 1 (3.0) | 0 | 0 |
| Nephritis | 0 | 0 | 2 (5.3) | 1 (2.6) | 0 | 0 | 0 | 0 |
| Neutropenia | 0 | 0 | 1 (2.6) | 0 | 1 (3.0) | 0 | 0 | 0 |
| Neutrophil count decreased | 0 | 0 | 0 | 0 | 1 (3.0) | 0 | 0 | 0 |
| Pneumonia | 0 | 0 | 0 | 0 | 0 | 0 | 1 (14.3) | 0 |
| Pneumonitis | 0 | 0 | 0 | 0 | 1 (3.0) | 0 | 0 | 0 |
| Renal failure | 0 | 0 | 1 (2.6) | 0 | 0 | 0 | 0 | 0 |
| Stomatitis | 0 | 0 | 0 | 0 | 0 | 0 | 1 (14.3) | 0 |
| Urinary tract infection | 0 | 0 | 1 (2.6) | 1 (2.6) | 0 | 0 | 0 | 0 |
| Vomiting | 0 | 0 | 0 | 0 | 0 | 0 | 1 (14.3) | 0 |

AE, adverse event; ALT, Alanine aminotransferase; AST, Aspartate aminotransferase; CAN, canakinumab; CARBO, carboplatin; CIS, cisplatin; GEM, gemcitabine; PAC, paclitaxel; PEM, pemetrexed; SPARTA, spartalizumab.

**Supplementary Table S6:** Grade 3/4 AEs (≥10% of patients), requiring additional therapy by preferred term (safety analysis set)

| **Preferred term** | **Group A (SPARTA/GEM/CIS) N=33**  **n (%)** | **Group B (SPARTA/PEM/CIS) N=38**  **n (%)** | **Group C (SPARTA/CARBO/PAC) N=33**  **n (%)** | **Group E (SPARTA/CAN/PEM/CIS) N=7**  **n (%)** |
| --- | --- | --- | --- | --- |
| Neutropenia | 5 (15.2) | 6 (15.8) | 5 (15.2) | 0 |
| Anemia | 4 (12.1) | 4 (10.5) | 3 (9.1) | 2 (28.6) |
| Pulmonary embolism | 0 | 2 (5.3) | 4 (12.1) | 1 (14.3) |
| Hypophosphataemia | 1 (3.0) | 4 (10.5) | 0 | 1 (14.3) |
| Hypokalaemia | 0 | 4 (10.5) | 0 | 0 |
| Diabetes mellitus | 0 | 0 | 0 | 1 (14.3) |
| Cholecystitis | 0 | 0 | 0 | 1 (14.3) |
| Enterocolitis | 0 | 0 | 0 | 1 (14.3) |
| Perichondritis | 0 | 0 | 0 | 1 (14.3) |
| COPD | 0 | 0 | 0 | 1 (14.3) |

AE, adverse event; CAN, canakinumab; CARBO, carboplatin; CIS, cisplatin; COPD Chronic obstructive pulmonary disease; GEM, gemcitabine; PAC, paclitaxel; PEM, pemetrexed; SPARTA, spartalizumab.

**Supplementary Table S7:** Adverse events of special interest (in ≥10% of patients) (safety analysis set)

| **Safety topic** | **Group A  (SPARTA/GEM/CIS)  N=33** | | **Group B  (SPARTA/PEM/CIS)  N=38** | | **Group C  (SPARTA/CARBO/PAC)  N=33** | | **Group E  (SPARTA/CAN/PEM/CIS) N=7** | |
| --- | --- | --- | --- | --- | --- | --- | --- | --- |
|  | **All grades n (%)** | **Grade ≥3 n (%)** | **All grades n (%)** | **Grade ≥3 n (%)** | **All grades n (%)** | **Grade ≥3 n (%)** | **All grades n (%)** | **Grade ≥3 n (%)** |
| **Spartalizumab** |  |  |  |  |  |  |  |  |
| Rash | 17 (51.5) | 0 | 20 (52.6) | 1 (2.6) | 22 (66.7) | 0 | 1 (14.3) | 0 |
| Nephritis | 6 (18.2) | 0 | 16 (42.1) | 4 (10.5) | 3 (9.1) | 0 | 3 (42.9) | 0 |
| Colitis/diarrhea | 8 (24.2) | 0 | 12 (31.6) | 3 (7.9) | 14 (42.4) | 2 (6.1) | 2 (28.6) | 1 (14.3) |
| Type 1 diabetes mellitus | 1 (3.0) | 0 | 8 (21.1) | 0 | 2 (6.1) | 0 | 1 (14.3) | 1 (14.3) |
| Peripheral nerve disorder | 4 (12.1) | 0 | 6 (15.8) | 0 | 11 (33.3) | 0 | 0 | 0 |
| Hyperthyroidism | 4 (12.1) | 0 | 5 (13.2) | 0 | 1 (3.0) | 0 | 0 | 0 |
| Hypothyroidism | 3 (9.1) | 0 | 5 (13.2) | 0 | 4 (12.1) | 0 | 0 | 0 |
| Pneumonitis | 4 (12.1) | 0 | 2 (5.3) | 0 | 4 (12.1) | 0 | 0 | 0 |
| **Canakinumab** | - | - | - | - | - | - |  |  |
| Infections | - | - | - | - | - | - | 5 (71.4) | 2 (28.6) |
| Opportunistic infections | - | - | - | - | - | - | 1 (14.3) | 0 |
| Thrombocytopenia | - | - | - | - | - | - | 1 (14.3) | 0 |

CAN, canakinumab; CARBO, carboplatin; CIS, cisplatin; GEM, gemcitabine; PAC, paclitaxel; PEM, pemetrexed; SAE, serious adverse event; SPARTA, spartalizumab.

**Supplementary Table S8:** PFS, OS, DOR and TTR per investigator's assessment (full analysis set)

|  | **Group A**  **(SPARTA/GEM/CIS)**  **N=33** | **Group B**  **(SPARTA/PEM/CIS)**  **N=38** | **Group C**  **(SPARTA/CARBO/PAC)**  **N=33** | **Group E**  **(SPARTA/CAN/PEM/CIS)**  **N=7** |
| --- | --- | --- | --- | --- |
| **PFS** |  |  |  |  |
| Evaluable patients, n (%) | 27 (81.8) | 25 (65.8) | 31 (93.9) | 7 (100) |
| Median follow-up, months | 6.14 | 9.03 | 6.11 | 7.52 |
| Median PFS, months (95% CI) | 6.2 (4.2-8.7) | 10.4 (5.4-26.4) | 6.3 (4.1-10.2) | 7.5 (4.1-12.4) |
| Estimated PFS rate,  % (95% CI) at |  |  |  |  |
| 6 months | 60.5 (41.8-74.8) | 63.1 (45.1-76.7) | 57.0 (38.4-71.9) | 71.4 (25.8-92.0) |
| 12 months | 24.5 (11.2-40.5) | 48.5 (31.2-63.7) | 25.3 (12.0-41.1) | 28.6 (4.1-61.2) |
| 24 months | 16.8 (5.9-32.4) | 36.1 (20.5-51.9) | 15.8 (5.8-30.4) | 0.0 (NE-NE) |
| **OS** |  |  |  |  |
| Evaluable patients, n (%) | 23 (69.7) | 22 (57.9) | 26 (78.8) | 6 (85.7) |
| Median follow-up, months | 15.97 | 28.62 | 15.31 | 20.96 |
| Median OS, months (95% CI) | 16.1 (10.0-21.7) | 29.7 (17.8-39.9) | 17.6 (9.4-23.3) | 21.0 (4.8-NE) |
| Estimated OS rate,  % (95% CI) at |  |  |  |  |
| 6 months | 97.0 (80.4-99.6) | 83.8 (67.4-92.4) | 84.7 (67.1-93.4) | 85.7 (33.4-97.9) |
| 12 months | 65.7 (46.7-79.3) | 75.4 (58.0-86.4) | 59.6 (40.8-74.2) | 57.1 (17.2-83.7) |
| 24 months | 32.5 (17.0-49.1) | 61.4 (43.7-75.1) | 31.4 (16.5-47.5) | 42.9 (9.8-73.4) |
| 36 months | 21.7 (7.6-40.4) | 47.0 (30.2-62.2) | 18.8 (7.6-33.8) | 0.0 (NE-NE) |
| 42 months | NE (NE-NE) | 29.9 (12.7-49.4) | 18.8 (7.6-33.8) | 0.0 (NE-NE) |
| **DOR** |  |  |  |  |
| Patients with a response | **N=19** | **N=21** | **N=17** | **N=4** |
| Evaluable patients, n (%) | 14/19 (73.7) | 11/21 (52.4) | 15/17 (88.2) | 3/4 (75.0) |
| Median follow-up, months | 5.03 | 13.83 | 7.39 | 3.63 |
| Median DOR, months (95% CI) | 6.0 (3.0-18.0) | 30.1 (9.0-NE) | 8.2 (5.1-23.1) | 7.1 (1.4-NE) |
| Estimated DOR rate,  % (95% CI) at |  |  |  |  |
| 6 months | 52.1 (28.0-71.6) | 76.2 (51.9-89.3) | 68.8 (40.5-85.6) | 50.0 (5.8-84.5) |
| 12 months | 34.7 (14.5-56.0) | 60.2 (35.7-77.9) | 31.3 (11.4-53.6) | 0.0 (NE-NE) |
| 24 months | 20.8 (5.6-42.7) | 54.7 (30.7-73.5) | 25.0 (7.8-47.2) | 0.0 (NE-NE) |
| **TTR** |  |  |  |  |
| Evaluable patients, n (%) | 19 (57.6) | 21 (55.3) | 17 (51.5) | 4 (57.1) |
| Median follow-up, months | 2.73 | 2.76 | 3.91 | 4.96 |
| Median TTR, months (95% CI) | 2.7 (1.3-NE) | 6.2 (1.4-NE) | 3.9 (1.3-NE) | 5.0 (1.3-NE) |
| Estimated TTR rate,  % (95% CI) at |  |  |  |  |
| 6 months | 45.5 (28.2-61.2) | 52.4 (35.0-67.2) | 48.5 (30.8-64.1) | 42.9 (9.8-73.4) |
| 12 months | 42.2 (25.3-58.2) | 46.6 (29.7-61.8) | 48.5 (30.8-64.1) | 42.9 (9.8-73.4) |
| 24 months | 42.2 (25.3­-58.2) | 40.7 (24.6-56.2) | 48.5 (30.8-64.1) | 42.9 (9.8-73.4) |
| 42 months | 42.2 (25.3-58.2) | 40.7 (24.6-56.2) | 48.5 (30.8-64.1) | 42.9 (9.8-73.4) |

CAN, canakinumab; CARBO, carboplatin; CI, confidence interval; CIS, cisplatin; DOR, duration of response; GEM, gemcitabine; NE, not evaluated; OS, overall survival; PAC, paclitaxel; PEM, pemetrexed; PFS, progression-free survival; SPARTA, spartalizumab; TTR, time to response.

**Supplementary Table S9:** Antitumor response by the PD-L1 subgroups

| **Best overall response, n (%)** | **PD-L1 expression in tumor cells (%)** | | | |
| --- | --- | --- | --- | --- |
|  | **<1%** | **≥1% to <50%** | **≥50%** | **Missing** |
| Group A (N=33) | n=9 | n=15 | n=7 | n=2 |
| CR | 0 | 0 | 1 (14.3) | 0 |
| PR | 3 (33.3) | 10 (66.7) | 3 (42.9) | 2 (100) |
| SD | 5 (55.6) | 3 (20.0) | 3 (42.9) | 0 |
| PD | 1 (11.1) | 2 (13.3) | 0 | 0 |
| ORR (CR+PR), n (%; 95% CI) | 3 (33.3; 7.5-70.1) | 10 (66.7; 38.4-88.2) | 4 (57.1; 18.4-90.1) | 2 (100; 15.8-100) |
| DCR (CR+PR+SD), n (%; 95% CI) | 8 (88.9; 51.8-99.7) | 13 (86.7; 59.5-98.3) | 7 (100; 59.0-100) | 2 (100; 15.8-100) |
| Group B (N=38) | n=9 | n=14 | n=6 | n=9 |
| CR | 1 (11.1) | 1 (7.1) | 0 | 0 |
| PR | 3 (33.3) | 7 (50.0) | 5 (83.3) | 4 (44.4) |
| SD | 1 (11.1) | 6 (42.9) | 1 (16.7) | 2 (22.2) |
| PD | 3 (33.3) | 0 | 0 | 1 (11.1) |
| Unknown | 1 (11.1) | 0 | 0 | 2 (22.2) |
| ORR (CR+PR), n (%; 95% CI) | 4 (44.4; 13.7-78.8) | 8 (57.1; 28.9-82.3) | 5 (83.3; 35.9-99.6) | 4 (44.4; 13.7-78.8) |
| DCR (CR+PR+SD), n (%; 95% CI) | 5 (55.6; 21.2-86.3) | 14 (100; 76.8-100) | 6 (100; 54.1-100) | 6 (66.7; 29.9-92.5) |
| Group C (N=33) | n=8 | n=14 | n=5 | n=6 |
| CR | 0 | 1 (7.1) | 1 (20.0) | 0 |
| PR | 4 (50.0) | 5 (35.7) | 2 (40.0) | 4 (66.7) |
| SD | 3 (37.5) | 5 (35.7) | 1 (20.0) | 1 (16.7) |
| PD | 1 (12.5) | 2 (14.3) | 0 | 1 (16.7) |
| Unknown | 0 | 1 (7.1) | 1 (20.0) | 0 |
| ORR (CR+PR), n (%; 95% CI) | 4 (50.0; 15.7-84.3) | 6 (42.9; 17.7-71.1) | 3 (60.0; 14.7-94.7) | 4 (66.7; 22.3-95.7) |
| DCR (CR+PR+SD), n (%; 95% CI) | 7 (87.5; 47.3-99.7) | 11 (78.6; 49.2-95.3) | 4 (80.0; 28.4-99.5) | 5 (83.3; 35.9-99.6) |
| Group E (N=7) | n=4 | n=2 | n=1 | n=0 |
| PR | 2 (50.0) | 1 (50.0) | 1 (100) | 0 |
| SD | 2 (50.0) | 1 (50.0) | 0 | 0 |
| ORR (CR+PR), n (%; 95% CI) | 2 (50.0; 6.8-93.2) | 1 (50.0; 1.3-98.7) | 1 (100; 2.5-100) | 0 (NA) |
| DCR (CR+PR+SD), n (%; 95% CI) | 4 (100; 39.8-100) | 2 (100; 15.8-100) | 1 (100; 2.5-100) | 0 (NA) |
| Median PFS, months (95% CI) |  |  |  |  |
| Group A | 6.2 (1.3-8.7) | 6.1 (4.0-7.9) | NE (4.0-NE) | 4.7 (4.3-NE) |
| Group B | 7.3 (1.1-NE) | 15.3 (4.1-NE) | 31.5 (3.9-NE) | 5.5 (0.8-10.4) |
| Group C | 6.2 (1.6-10.2) | 5.2 (1.3-12.3) | 12.8 (2.1-NE) | 7.1 (1.3-NE) |
| Group E | 7.8 (4.1-NE) | 6.0 (4.8-NE) | 12.4 (NE-NE) | - |

CI, confidence interval; CR, complete response; DCR, disease control rate; NA, not available; NE, not evaluated; ORR, overall response rate; PD, progressive disease; PD-L1, programmed cell-death ligand 1; PFS, progression-free survival; PR, partial response; SD, stable disease.

**Supplementary Table S10:** Pharmacokinetic parameters for spartalizumab by group (pharmacokinetic analysis set)

| **Parameter** | **Visit** | **Statistics** | **Group A**  **(SPARTA/GEM/CIS) N=33** | **Group B**  **(SPARTA/PEM/CIS) N=38** | **Group C**  **(SPARTA/CARBO/PAC) N=33** | **Group E**  **(SPARTA/CAN/PEM/CIS) N=7** |
| --- | --- | --- | --- | --- | --- | --- |
| AUC_last_ (h*µg/mL) | C1 D1 | n | 29 | 37 | 33 | 6 |
|  |  | Geo-mean | 13500 | 15200 | 15300 | 15900 |
|  |  | Geo-CV% | 25.8 | 35.0 | 31.3 | 26.3 |
|  | C3 D1 | n | 27 | 30 | 26 | 6 |
|  |  | Geo-mean | 25200 | 26600 | 21000 | 28700 |
|  |  | Geo-CV% | 31.0 | 40.9 | 58.7 | 52.0 |
|  | C4 D1 | n | 26 | 26 | 21 | 7 |
|  |  | Geo-mean | 24900 | 31500 | 23900 | 27300 |
|  |  | Geo-CV% | 48.5 | 48.7 | 46.2 | 52.4 |
| C_trough_ (µg/mL) | C1 D1 | n | 24 | 29 | 30 | 7 |
|  |  | Geo-mean | 15.6 | 18.4 | 15.4 | 19.9 |
|  |  | Geo-CV% | 27.2 | 34.6 | 71.7 | 36.3 |
|  | C3 D1 | n | 22 | 25 | 18 | 5 |
|  |  | Geo-mean | 37.2 | 34.8 | 35.4 | 46.7 |
|  |  | Geo-CV% | 29.8 | 52.6 | 39.0 | 17.8 |
|  | C4 D1 | n | 22 | 24 | 19 | 6 |
|  |  | Geo-mean | 42.2 | 50.8 | 39.7 | 46.1 |
|  |  | Geo-CV% | 31.3 | 28.4 | 41.5 | 41.1 |
| C_max_ (µg/mL) | C1 D1 | n | 29 | 37 | 33 | 6 |
|  |  | Geo-mean | 59.7 | 62.9 | 64.7 | 68.2 |
|  |  | Geo-CV% | 26.9 | 30.8 | 37.0 | 24.7 |
|  | C3 D1 | n | 27 | 30 | 26 | 6 |
|  |  | Geo-mean | 84.5 | 90.4 | 81.1 | 89.5 |
|  |  | Geo-CV% | 30.2 | 33.0 | 28.4 | 30.4 |
|  | C4 D1 | n | 26 | 26 | 21 | 7 |
|  |  | Geo-mean | 96.0 | 104 | 93.7 | 88.8 |
|  |  | Geo-CV% | 20.8 | 28.5 | 34.0 | 45.5 |
| T_max_ (hr) | C1 D1 | n | 29 | 37 | 33 | 6 |
|  |  | Median | 0.567 | 0.767 | 0.7 | 0.617 |
|  |  | Min-Max | 0.0-167 | 0.5-172 | 0.45-2.50 | 0.55-2.63 |
|  | C3 D1 | n | 27 | 30 | 26 | 6 |
|  |  | Median | 0.633 | 0.667 | 0.683 | 0.558 |
|  |  | Min-Max | 0.467-337 | 0.467-2.38 | 0.333-168 | 0.467-0.750 |
|  | C4 D1 | n | 26 | 26 | 21 | 7 |
|  |  | Median | 0.850 | 0.975 | 0.750 | 1.00 |
|  |  | Min-Max | 0.00-24.2 | 0.533-146 | 0.467-23.0 | 0.5-145 |

AUC, The area under the concentration-time curve; C, cycle; CAN, canakinumab; CARBO, carboplatin; CIS, cisplatin; C_max_, maximum or peak serum concentration; C_trough_, trough plasma concentration; CV, coefficient of variation; D, day; Max, maximum; Min, minimum; GEM, gemcitabine; Geo, geometric; PAC, paclitaxel; PEM, pemetrexed; SPARTA, spartalizumab; T_max_, time to reach the maximum concentration.

**Supplementary Table S11:** Pharmacokinetic parameters for canakinumab in group E (pharmacokinetic analysis set)

| **Parameter** | **Statistics** | **I-C1D1 N=7** |
| --- | --- | --- |
| AUC_last_ (h*ng/mL) | n | 7 |
|  | Geo-mean | 5970000 |
|  | Geo-CV% | 46.6 |
| C_trough_ (ng/mL) | n | 7 |
|  | Geo-mean | 11500 |
|  | Geo-CV% | 40.0 |
| C_max_ (ng/mL) | n | 7 |
|  | Geo-mean | 16700 |
|  | Geo-CV% | 46.4 |
| T_max_ (hr) | n | 7 |
|  | Median | 168 |
|  | Min-Max | 144-336 |

AUC, area under the concentration curve; C1D1, day 1 of cycle 1; C_max_, maximum or peak serum concentration; CV, coefficient of variation; Geo, geometric; T_max_, time to reach the maximum concentration
